# Systematic review and meta-analysis of oral squamous cell carcinoma associated oral microbiome

**DOI:** 10.3389/fmicb.2022.968304

**Published:** 2022-10-20

**Authors:** Tabitha K. Peter, Miyuraj H. H. Withanage, Carissa L. Comnick, Chandler Pendleton, Shareef Dabdoub, Sukirth Ganesan, David Drake, Jeffrey Banas, Xian Jin Xie, Erliang Zeng

**Affiliations:** ^1^Division of Biostatistics and Computational Biology, College of Dentistry, University of Iowa, Iowa City, IA, United States; ^2^College of Dentistry, Iowa Institute for Oral Health Research, University of Iowa, Iowa City, IA, United States; ^3^Department of Periodontics, College of Dentistry, University of Iowa, Iowa City, IA, United States; ^4^Department of Pediatric Dentistry, College of Dentistry, University of Iowa, Iowa City, IA, United States; ^5^Department of Preventative and Community Dentistry, College of Dentistry, University of Iowa, Iowa City, IA, United States

**Keywords:** oral cancer, oral microbiome, oral squamous cell carcinoma, oral health, *Fusobacterium*, ABC transport, data collecting, meta-analysis

## Abstract

The intersection between the human oral microbiome and oral health is an emerging area of study which has gained momentum over the last decade. This momentum has motivated a search for associations between the oral microbiome and oral cancer, in hopes of identifying possible biomarkers that facilitate earlier diagnosis and improved prognosis for patients with that disease. The present study examined the relationship between the microbiome in the human oral cavity and oral squamous cell carcinoma (OSCC). We searched the literature for case-control studies which focused on the relationship between the human oral microbiome and OSCC. We aggregated three types of data from these studies: bacteriome data at the genus level, predicted functional pathway data, and gene abundance data. From these data, we noted several microbial genera which may be associated with oral cancer status, including *Fusobacterium*. We also identified functional pathways which merit further investigation, including RNA degradation (ko03018) and primary immunodeficiency (ko05340). In addition, our analysis of gene abundance data identified the gene K06147 (ATP-binding cassette, subfamily B, bacterial) as being over abundant in OSCC samples. Our results are generalizations which identified some currents that we believe could guide further research. Our work faced several limitations related to the heterogeneity of the available data. Wide variation in methods for sample collection, methods for controlling for known behavioral risk factors, computing platform choice, and methods for case-control design all posed confounding factors in this work. We examined the current methods of data collection, data processing, and data reporting in order to offer suggestions toward the establishment of best practices within this field. We propose that these limitations should be addressed through the implementation of standardized data analytic practices that will conform to the rigor and reproducibility standards required of publicly funded research.

## Introduction

Oral squamous cell carcinoma (OSCC) is among the most common types of head and neck cancer, and it is the cancer with the highest incidence rate among South-Central Asian populations (Irfan et al., 2020; Ferlay et al., 2021). Data from recent years indicate that the incidence of this disease is increasing in some populations, and the 5-year survival rate remains around 50% worldwide (Garćıa-Mart́ın et al., 2019). Previous investigations of OSCC have established connections between the disease and well-known behavioral risk factors, including tobacco use and alcohol consumption (Coletta et al., 2020). However, OSCC continues to present in patients who have not been exposed to these behavioral risks. Contemporary research on OSCC investigates interactions with and influences of the human oral microbiome, with the goal of providing insight into the etiology of OSCC.

As the amount literature on OSCC and the human microbiome (especially the bacteriome) has increased, so has the number of approaches for studying these interactions and influences at the taxonomic, functional, and genetic levels (Doud et al., 2009; Zhang et al., 2014; Aguiar-Pulido et al., 2016; Sedghi et al., 2021). Moreover, results shown to be “significant” have notable variation in the existing literature. For instance, two case-control studies of OSCC have reported significant relationships between alpha diversity and OSCC samples, but with associations in opposite directions (Guerrero-Preston et al., 2016; Zhao et al., 2017). This dissonance has also appeared in comparisons of the relative abundance of specific genera (and/or species) between OSCC and control samples. As an example, the genus *Streptococcus* has been found significantly differentially abundant among both OSCC samples (Zhou et al., 2020) and control samples (Schmidt et al., 2014; Zhao et al., 2017; Zhang et al., 2020).

In addition to the dissonance between findings in this field, choices of sample collection methods and study designs have shown wide variation. Some studies have examined only samples from a specific location in the oral cavity, such as the tongue (Mukherjee et al., 2017) or the buccal mucosa (Su et al., 2021), while others have been so inclusive as to examine samples representing the oral cavity, pharynx, and/or larynx (Schmidt et al., 2014; Börnigen et al., 2017; Hayes et al., 2018). The influences of this variation in site have been further compounded by heterogeneity in the methods used for sample collection, which have included oral rinse samples (Börnigen et al., 2017), fresh-frozen tissue samples (Mukherjee et al., 2017), and oral swabs (Mok et al., 2017). Further compounding these challenges is a lack of coherence in the inclusion/exclusion criteria used for patient recruitment with regard to behavioral risk factors (e.g., tobacco and alcohol consumption). Although such behavioral factors have been well-established as risks for oral cancer (Abati et al., 2020), some authors have been imprecise in reporting how behavioral risk factors impact patient recruitment or confound patterns in the OSCC-microbiome relationship. Generalizations regarding both biological associations and best analytic practices are needed to improve the development, rigor, and reproducibility of results in this rapidly evolving field.

The need for such generalizations has been echoed in a recent systematic review by Su Mun et al. (2021). This review provided a qualitative summary of the literature regarding OSCC-microbiome associations and noted the heterogeneity of both the methods and results found in this body of literature. In the present study, we investigated the relationship between the human oral bacteriome and OSCC using taxonomic, functional, and gene abundance data aggregated from multiple published studies. Our objective was twofold; first, we aimed to make quantitative generalizations about previously posited connections between the human oral bacteriome and OSCC. Through a literature search and meta-analysis of case-control studies with OSCC patients, we identified bacterial genera, functional pathways, and genes which may be associated with OSCC status. Our second aim was to assess this emerging field of research from a data-analytic perspective. We made specific suggestions toward the establishment of norms in data collection, analysis, and reporting, as this would increase the robustness of the results and facilitate the comparison and aggregation of data across studies.

## Methods

###  Systematic literature search

We began our systematic literature search by searching the PubMed database. Our literature search keywords “(oral cancer OR mouth cancer) AND (bacteria OR bacterium OR microbiome)” returned nearly 140 abstracts published before July 31, 2020. To distill these into a well-defined set of studies for further review, we established a set of criteria. Each paper that we included in our systematic review and meta-analysis met these criteria:

1. Available in English and report on experiment-based results (i.e., not a case report or a literature review).
2. Pertained to cancer of the oral cavity. Studies that included samples from the throat area (e.g., oropharynx) were considered in the review only (not in the meta-analysis)^1^.
3. Reported on human patient research (i.e., no cell line models or animal models) were permitted.
4. Reported on a study which had a case-control design and an objective of differentiating between the oral microbiome of the OSCC case samples and that of cancer-free samples. We chose to exclude studies of cancer progression (e.g., studies with a focus on comparing OSCC across various stages of disease).

**Figure 1:**
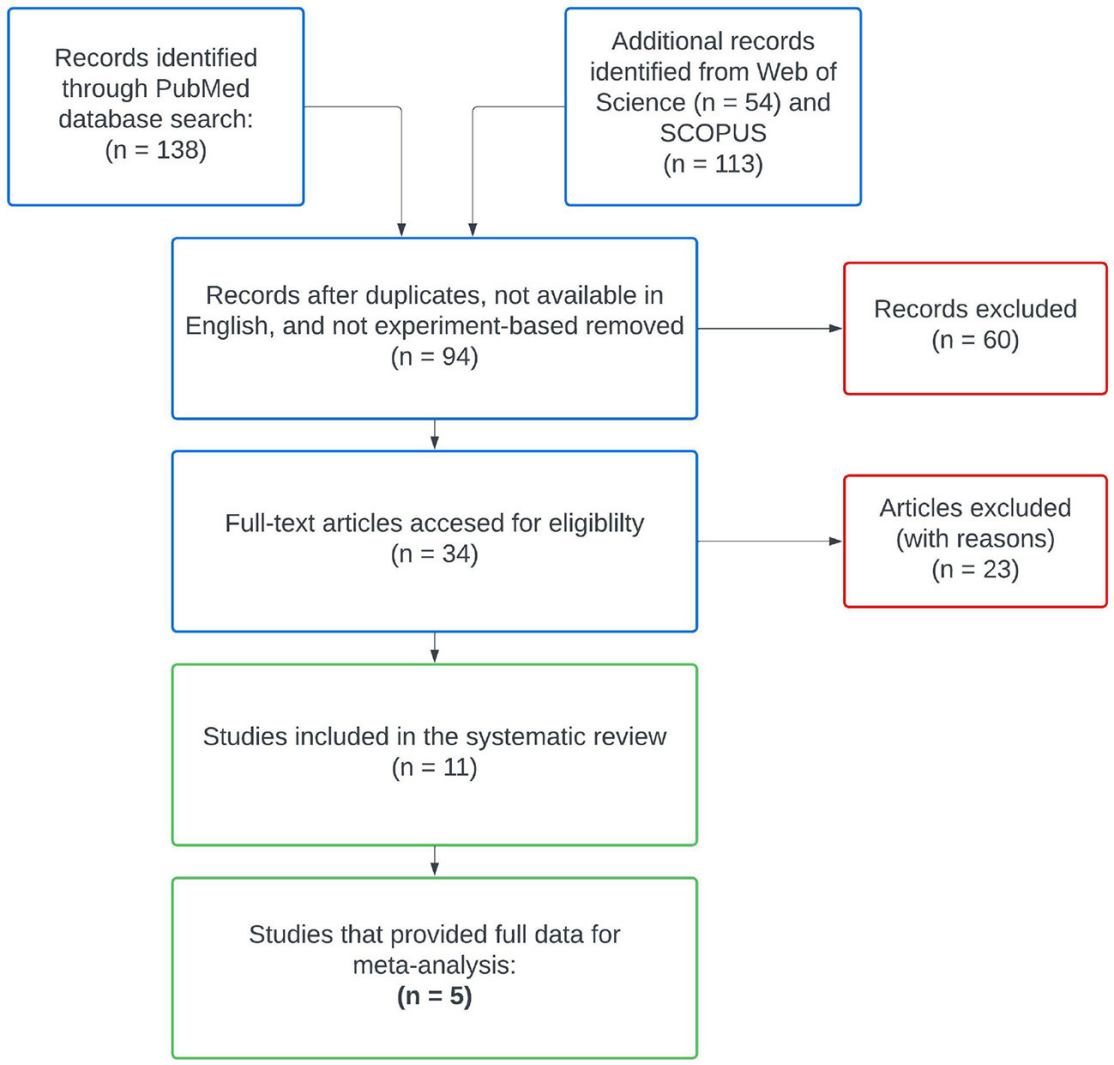
PRISMA diagram for the systematic literature search. Only five studies met all of our inclusion criteria for meta-analysis and supplied publicly available data sets.

After reviewing abstracts returned by PubMed, we searched Web of Science and SCOPUS databases using the same keywords. Following aggregation and filtering all results through our criteria, eleven remaining studies were included in our literature review (Figure 1, Pan et al., 2014; Guerrero-Preston et al., 2016; Börnigen et al., 2017; Mok et al., 2017; Mukherjee et al., 2017; Zhao et al., 2017; Perera et al., 2018; Takahashi et al., 2019; Zhang et al., 2020; Zhou et al., 2020; Su et al., 2021). We subgrouped these eleven studies according to the type of control used in their study designs, differentiating between intra-subject control designs and inter-subject control designs. Intra-subject control design studies used healthy samples from the OSCC patients as the control group, whereas studies with an inter-subject control design used samples from an independent set of OSCC-free participants as the control group. From the 11 studies in our review, six reported on experiments with inter-subject designs and the remaining five reported on experiments with paired designs. We kept data from these subgroups separated throughout all analyses, as samples from differing control designs represent biologically different populations.

###  Description of clinical data available

The data from our eleven reviewed studies as show in Table 1 represented samples from 970 patients across seven different countries. All of these studies were published between 2013 and 2020 (Table 1). The average age of OSCC patients in these studies was ~61 years^2^. Assessing the impact of behavioral risk factors like smoking and drinking was not the focus of these studies; several authors indicated that patients who smoked or drank were included in their respective studies, and other authors left these inclusion criteria in ambiguity (Table 1).

**Table 1:** Description of patients/participants.

**References**	**Control type**	**Study population**	**Number of patients**	**Age (cases only)**	**Age (controls only)**	**Age measure (mean vs. median)**
Pan et al. (2014)	Other	China	128	68.10	70.20	
Guerrero-Preston et al. (2016)	Other	USA	42	64.00	NA	Median
Zhao et al. (2017)	Self	China	40	62.00	NA	Median
Mok et al. (2017)	Other	Malaysia	18	60.00	40.00	Mean
Mukherjee et al. (2017)	Self	USA	39	60.50	NA	Mean
Börnigen et al. (2017)	Other	USA	363	58.00	NA	Median
Perera et al. (2018)	Other	Sri Lanka	52	61.00	50.58	
Takahashi et al. (2019)	Other	Japan	140	63.70	65.10	Mean
Zhang et al. (2020)	Self	China	50	60.70	NA	Mean
Zhou et al. (2020)	Self	China	24	61.10	NA	Mean
Su et al. (2021)	Self	Taiwan	74	53.96	NA	Mean

Regarding the data type, 16S data representing the microbiome were pervasive. Given the available data, we also focused our meta-analysis on 16S data of bacterial specimens. Although other components of the microbiome, including the mycome and the virome, have also been studied in the etiology of oral cancer (Al-Hebshi et al., 2019; Di Cosola et al., 2021), these elements were beyond the scope of the present study.

Although all eleven studies reported quantitative results, the methods and format used in reporting varied. Most studies reported aggregated measurements (e.g., average relative abundance of a given genera across all samples from OSCC patients). Of the eleven studies reviewed, only those by Zhao et al. (2017), Perera et al. (2018), Takahashi et al. (2019), Zhang et al. (2020), and Zhou et al. (2020) provided sequencing data in a public data repository sufficient for inclusion in our meta-analysis. Our meta-analysis examined the taxonomic and functional profiles from the individual samples represented in these five publicly available data sets.

###  Taxonomic meta-analysis

**Figure 2:**
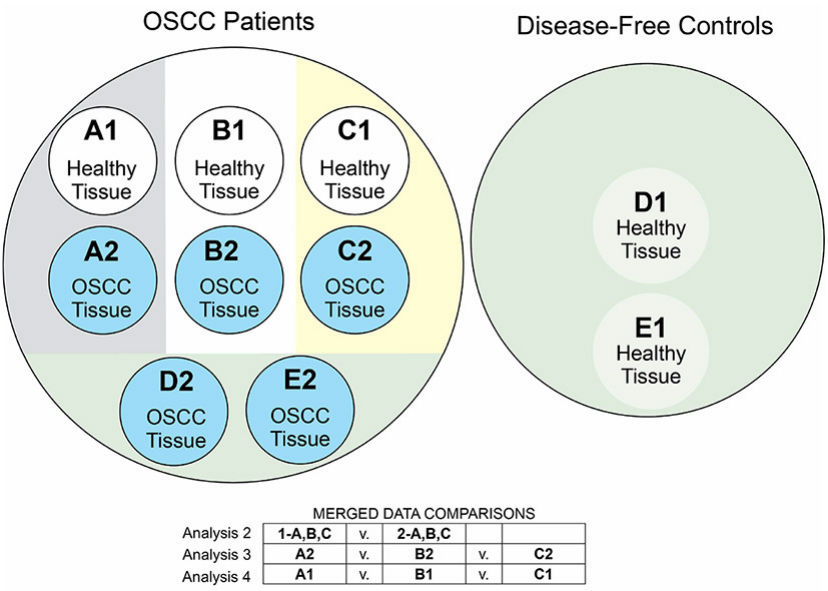
Diagram of meta-analytic comparisons. We labeled each of the five studies in our meta-analysis with letters A–E, where A-C indicated the three studies with intra-subject control designs. We labeled each set of samples with the numbers 1-2, indicating the control/OSCC status of those samples. In our analysis 1, we examined the OSCC case vs. control samples within each study, comparing A1 vs. A2, B1 vs. B2, C1 vs. C2, D1 vs. D2, and E1 vs. E2. Analysis 2 compared the merged A2 + B2 + C2 case samples with the A1 + B1 + C1 control samples. Analysis 3 compared the case samples A2 vs. B2 vs. C2. Analysis 4 compared the control samples A1 vs. B1 vs. C1. The circle diagram summarizes these comparisons.

Among the five studies that provided full data, Perera et al. (2018) and Takahashi et al. (2019) described an inter-subject control design while the others all described an intra-subject design. Recognizing that heterogeneity of samples may arise from factors apart from disease status (e.g., behavioral risk factors, study design, and sample collection method), our taxonomic analyses involved four distinct strategies (Figure 2). We examined these specific comparisons, each at the genus level:

1. Analysis of case vs. control samples within each of the five studies;
2. Analysis of merged case vs. merged control samples within data from all intra-subject controlled studies;
3. Analysis of case vs. case samples between all intra-subject controlled studies;
4. Analysis of control vs. control samples between all intra-subject controlled studies.

In Figure 2, letters A–E indicate the study and the numbers 1–2 indicate OSCC-free samples vs. OSCC diseased samples, respectively.

We used QIIME2 (Bolyen et al., 2019) to preprocess the data aggregated from the studies A, B, C, D, and E (Zhao et al., 2017; Perera et al., 2018; Takahashi et al., 2019; Zhang et al., 2020; Zhou et al., 2020), respectively. This preprocessing included removing genera with zero counts^3^. We also removed any genera not classified as Bacteria, and any genera with ambiguous names (e.g., “uncultured”). To normalize the data, we transformed the counts of genera into proportions by dividing the number of counts of a given genus in a sample by the total number of counts of all genera observed in that sample. This gave values for the abundance of each genus per each sample. To obtain information for each genus, we summed the per-genus counts across all samples. We refer to the resulting values as measures of normalized relative abundance.

In each of these four sub-analyses, we used the Shannon diversity (Shannon, 1948) index to quantify alpha diversity from the counts of each genus. These indices were compared using a Wilcoxon rank-sum test. Comparisons of normalized relative abundance at the genus level were done using the log-transformed ratios of median relative abundance of each genus using Wilcoxon rank-sum tests (analyses 1 and 2) and Kruskal-Wallis tests (analysis 3 and 4). All *p*-values are adjusted for multiple testing using FDR, and significance was set at a false discovery rate of 0.05. These data analytic methods were implemented using the “taxa” and “metacoder” packages in R (Foster et al., 2017, 2018) as modeled in the package vignettes.

In addition to these non-parametric statistical methods, we used the linear discriminant analysis effect size (LEfSe) method to identify the distinguishing taxa within the OSCC and control groups. The authors of this method have shown LEfSe has relatively low false positive rate and considers both the statistical significance and the effect size in determining microbiome-associated biomarkers (Segata et al., 2011). For the purposes of our work, LEfSe served as a conservative check of the taxonomic results identified by the other analytical methods.

###  Functional meta-analysis

Representative sequences of amplicon sequence variants (ASV) and the corresponding biom table of ASVs across samples were input into PICRUSt2 (Douglas et al., 2020). This allowed for the prediction of bacterial functional potential in terms of KEGG orthologs. KEGG ortholog annotation was downloaded in json format and annotated with a PICRUSt-predicted KEGG orthologs table (Kanehisa et al., 2021). With these results, a new table was created with counts for each item in third hierarchical level of the KEGG database file.

With the KEGG ortholog abundance values, we performed LEfSe analysis to identify ortholog functions that may act as potential biomarkers. As part of this analysis, we preprocessed our dataset to suit LEfSe's input data format. This analysis pipeline was used in each of the four specific comparisons as shown in Figure 2.

## Results

###  Analysis 1 (case vs. control)

The first component of our taxonomic meta-analysis re-examined the results of the five original studies using our data processing workflow and our analytic methods. The differences between our work and the previously published results provided evidence of the impact of preprocessing techniques on the results. This evidence is presented in Supplementary Figures 1–12.

###  Analysis 2 (merged case vs. merged control)

#### Taxonomic results

**Figure 3:**
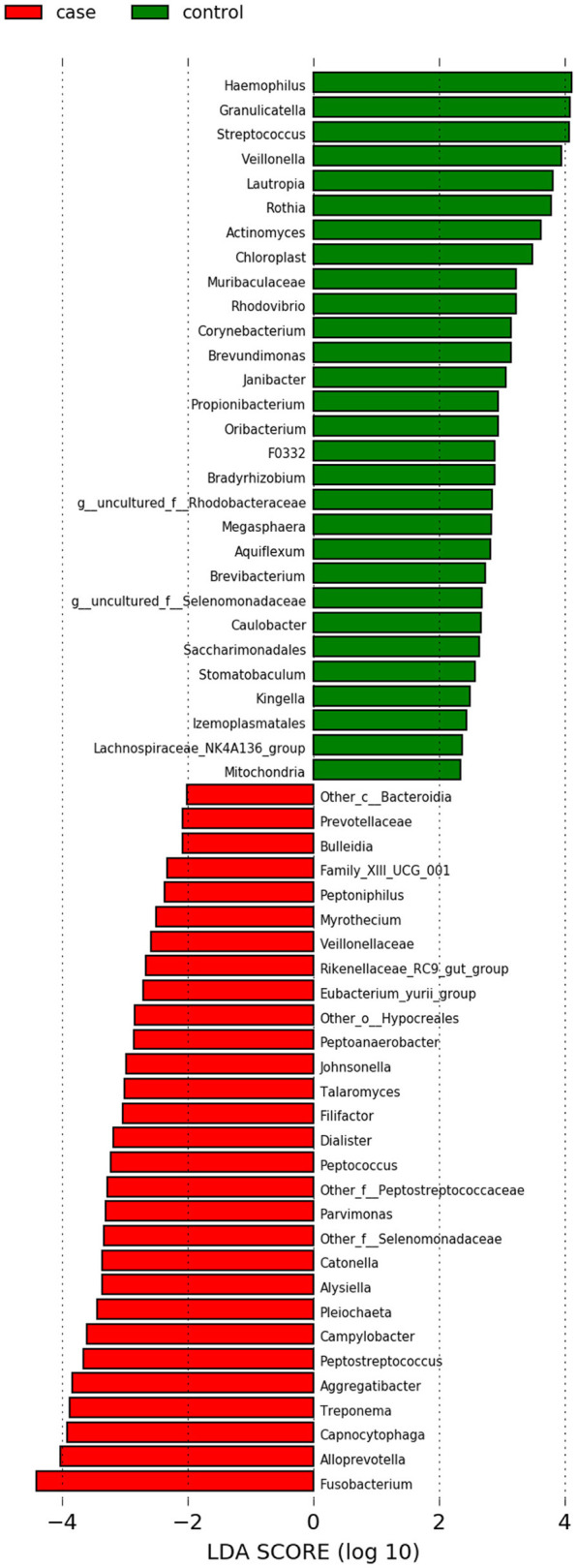
LEfSe results for the taxonomic analysis in Analysis 2 (case vs. control). When using the LDA score as a metric for comparison, *Fusobacterium* had the score of highest magnitude among the genera which were significantly differentially more abundant in case samples (shown in red).

In our comparison of the case versus control samples from the studies with an intra-control design, we found no evidence of a difference in alpha diversity as measured by the Shannon diversity index (Supplementary Figure 14). LEfSe results illustrated that the genera *Fusobacterium, Peptostreptococcus*, and *Parvimonas* appeared enriched in case samples, while *Haemophilus* and *Granulicatella* appeared enriched in the control samples (Figure 3). The Wilcoxon rank sum test results gave evidence of 20 genera showing a difference in relative abundance between case and control samples at a false discovery rate of 0.05 (Supplementary Figure 13). Eleven of which had a non-zero, finite estimate of effect size. All 11 of these Wilcoxon results were also represented in the LEfSe results.

#### Functional results

We found multiple potential functionalities involved in pathways relating to pyrimidine metabolism and RNA degradation were significantly upregulated among OSCC samples (effect size >3) (Supplementary Figure 15). Additionally, functions associated with the biosynthesis of vancomycin group antibiotics was upregulated in OSCC samples (effect size >2.5). The LEfSe analysis also showed that the functional pathways for (1) phenylalanine, tyrosine, and tryptophan (PTT) biosynthesis and (2) inositol phosphate metabolism were significantly downregulated in case samples (Supplementary Figure 15).

###  Analysis 3 (case vs. case)

#### Taxonomic results

To determine the effects of confounding factors on the observed microbial differences between OSCC and cancer-free sites, we compared the microbial composition and functions between OSCC samples from each of the intra-subject controlled studies. Data from Zhou et al. had a much lower alpha diversity than that of the other two studies (Supplementary Figure 18).

The Wilcoxon test identified over 80 genera as being significantly differentially abundant between the case samples from the Zhang, Zhao, and Zhou studies (Supplementary Data Set 1). Being a more conservative test, LEfSe identified only 32 genera to be significantly differentially abundant in this case vs. case comparison (Supplementary Figure 19). Our LEfSe analysis of the genera found that *Prevotella* and *Actinomyces* were enriched among samples from Zhao, which illustrated the heterogeneity of the case samples.

#### Functional results

A total of four pathways at KEGG level 3 were identified as being upregulated in the Zhou analysis (Supplementary Figure 20) with an effect size >3, including the inositol phosphate metabolism (ko00562). This simultaneously confounded the analysis 2 finding that inositol phosphate metabolism was upregulated among control samples and reinforced the observation that the Zhou data set had different characteristic from the other two data sets.

###  Analysis 4 (control vs. control)

#### Taxonomic results

In this taxonomic analysis, alpha diversity did not show evidence of variation between the control samples from the studies with an intra-subject control design (Supplementary Figure 21). Among these comparisons between the control samples, the Kruskal-Wallis test found evidence of 114 genera having significant differences in normalized relative abundance at a false discovery rate of 0.05 (Supplementary Data Set 2). The LEfSe results indicated that the strongest evidence of a difference appeared in the genera *Allorhizobium-Neorhizobium-Pararhizobium-Rhizobium, Prevotella*, and *Streptococcus* (Supplementary Figure 22).

These control vs. control results indicated that some of the most common ASVs in the oral cavity had high degrees of variation between the control samples in different studies. At the genus level, *Gemella, Granulicatella, Streptococcus*, and *Veillonella* have been identified as common in the human oral cavity (Aas et al., 2005). All of these except *Granulicatella* had a relatively high magnitude of discrimination among the aggregated control samples (Supplementary Figure 22). Moreover, several of the other genera with significant differentiation in relative abundance, including *Treponema, Prevotella, Selenomonas*, and *Capnocytophaga*, were among the genera with the highest counts in the taxonomic level data of the Human Oral Microbiome Database (HOMD) (Escapa et al., 2018).

The fact that the heterogeneity in control samples concerns the most common bacterial strains in the oral cavity may be attributable to differences in sampling methods and/or sample locations. Zhang and colleagues studied surface scrapes of buccal mucosa sites; Zhao and colleagues studied swabs of in different oral sites; and Zhou and colleagues studied tissue samples representing several oral sites. The control vs. control analysis provided results indicating that these control samples represent different definitions of a “normal” oral microbiome. For example, the “normal” microbiome of the buccal mucosa may be different from the “normal” microbiome of the mouth floor, and the “normal” oral tissue sample likely has a different microbial makeup compared to a “normal” oral swab. Such evidence suggests that the dysbiotic oral microbiome effects associated with OSCC may be site-specific, affecting specific areas of the mouth in different ways. This evidence also has implications for the standardization of sampling methods and the specification of sample inclusion criteria. We return to these points in the Discussion.

#### Functional results

A total of 11 pathways were upregulated in either the Zhang or Zhou studies (Supplementary Figure 23). We noticed that several of the pathways which appeared significant in analysis 2 appeared here also, which is an indication of confounding. Pyrimidine metabolism (ko00240) and PTT biosynthesis (ko00400) both appeared upregulated in the Zhang data.

###  Synthesis of results

**Figure 4:**
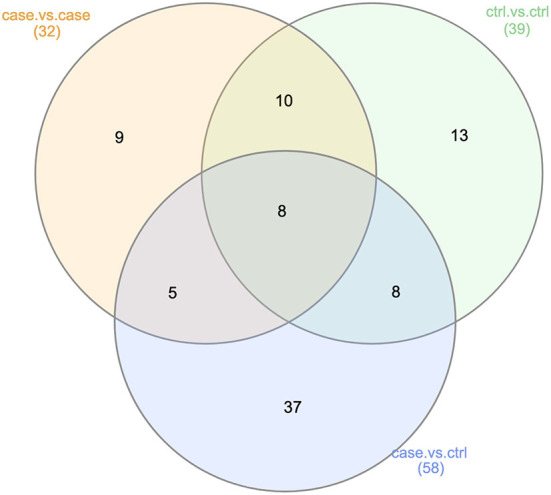
Summary of taxonomic LEfSe results (Analyses 2, 3, and 4). This Venn diagram describes the extent of the confounding represented in our case vs. control taxonomic analysis (Analysis 2). Of the 58 genera found to be significantly differentially abundant in either the OSCC or control samples, 21 were also found to be significantly differentially abundant in the case vs. case or control vs. control comparisons. This was an indication of non-OSCC related differences between the samples from studies A, B, and C.

**Figure 5:**
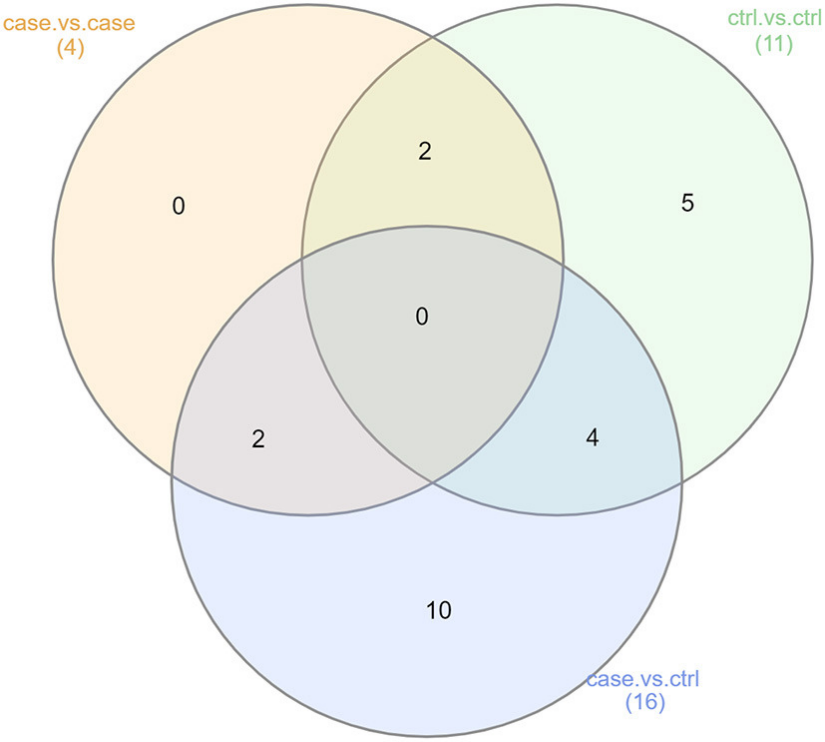
Summary of functional LEfSe results (Analyses 2, 3, and 4). This Venn diagram describes the extent of confounding represented in our case vs. control functional analysis (Analysis 2). Of the 16 functional pathways found to be significantly more abundant in either the OSCC or control samples, 6 were also showed significant differences in abundance in either the case vs. case or control vs. control comparisons. This was another indication of non-OSCC related differences between the samples from studies A, B, and C.

We summarized the taxonomic and functional results from analyses 2, 3, and 4 using Venn diagrams (Figures 4, 5). A total of 37 genera were identified as significantly differentially abundant in the case vs. control comparisons alone, including Fusobacterium (increased in OSCC samples), Haemophilus (decreased in control samples), and Granulicatella (decreased in case samples; Supplementary Data Set 3). A total of 10 functional pathways were identified as significantly differentially abundant in the case vs. control comparisons alone. Primary immunodeficiency (ko05340), plant pathogen interaction (ko04626), and RNA degradation (ko03018) were all upregulated among OSCC samples, whereas glutathione metabolism (ko00480), ubiquinone biosynthesis (ko00130), biosynthesis of unsaturated fatty acids (ko01040), Cushing syndrome (ko04934), regulation of actin cytoskeleton (ko04810), cell adhesion molecules (ko04514), and mineral absorption (ko04978) were all downregulated among OSCC samples (Supplementary Data Set 4).

**Figure 6:**
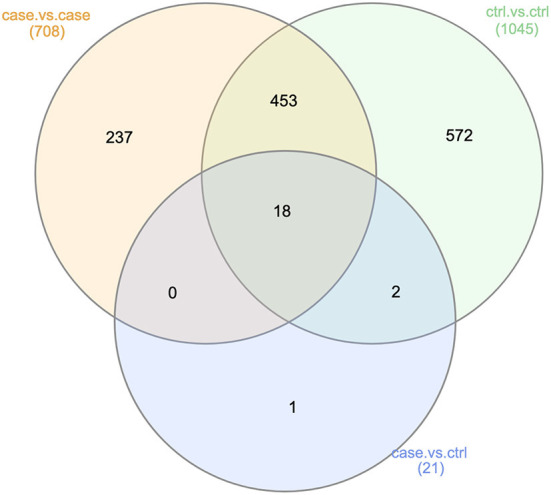
Genes found significant across all analyses. This Venn diagram describes the extent of confounding represented in our case vs. control analysis at the gene level (Analysis 2). Of the 21 genes found to be significantly differentially abundant in either the OSCC or control samples, 20 were also found to be significantly differentially abundant in either the case vs. case or control vs. control comparisons. This was another indication of non-OSCC related differences between the samples from studies A, B, and C.

Having considered integrating taxonomic results of case vs. control, case vs. case, and control vs. control comparisons, we were interested to investigate whether any results at the gene level were significant in only the case vs. control comparison. The Venn diagram in Figure 6 illustrated that among 1,313 genes examined, only one was found to be significant in only the case vs. control comparison. This was the gene with KEGG term K06147, an ATP-binding cassette (ABC—subfamily B, bacterial).

## Analysis of results and discussion

###  OSCC associated pathogenic bacteria

Enrichment of ASVs from the *Fusobacterium* genus in OSCC samples in our analysis was in line with the other findings, both within and outside the oral cavity. *Fusobacterium nucleatum* has been found to be an active bacterium in promoting oral cancer by several mechanisms (McIlvanna et al., 2021; Zhang S. et al., 2021; Liu et al., 2022). *F. nucleatum* has also been traditionally associated with chronic inflammation, the promotion of EMT among epithelial cells, the alteration of immune response in the oral cavity, and significant roles in several oral diseases including oral cancer and endodontic infections (Shao et al., 2021). Additionally, *F. nucleatum* and other gram-negative species have been found to show strong positive correlation with oral mucositis, a painful side effect common among patients undergoing chemotherapy (Hong et al., 2019). Outside the oral cavity, *F. nucleatum* has been found to be associated with pancreatic cancer, hepatic cancer, and breast cancer (Irfan et al., 2020) as well as human colorectal carcinoma (Castellarin et al., 2012; Nakatsu et al., 2015; Osman et al., 2021; Löwenmark et al., 2022). Evidence from our results suggested that bacteria from the *Fusobacterium* genus may be considered an oral carcinogenic bacteria. This generalization aligned with the observations reported in the recent reviews by Su Mun et al. (2021) and Alon-Maimon et al. (2022).

The findings from our work also connected to the literature of periodontal disease. Periodontitis has been posited as a risk factor for oral cancer (Komlós et al., 2021). In connection with the preceding paragraph, species from *Fusobacterium* have been identified as having associations with periodontitis (Mohanty et al., 2019). In particular, Han and colleagues recently reported that *F. nucleatum* secretes an amyloid-like adhesin which enhances its pathogenicity and promotes periodontal bone loss (Meng et al., 2021). Our meta-analysis work re-iterates the plausibility that bacteria associated with periodontal disease also contribute to the etiology of OSCC.

###  OSCC and connections to specific genes

The gene K06147, which was the sole gene significantly differentiated between OSCC and control samples in this work, is an ABC transporter. ABC transporters have been noted for two roles: the import essential nutrients and the export of toxic molecules (Davidson et al., 2008). They have been found to be distributed in all three kingdoms of living organisms and contribute to drug resistance (Lage, 2003). The results of our meta-analysis indicated that bacteria ABC transporters could be associated with human oral cancer. However, the mechanism of crosstalk between bacteria and host was not yet clear and needs further investigation. This gene has also been found to be enriched in lung cancer broncho-alveolar lavage fluid (BALF) samples (Zhang M. et al., 2021). In contrast, a study comparing the gut microbiota of colorectal cancer patients and health controls found that K06147 was significantly enriched in the control samples (Ai et al., 2019).

###  OSCC associated functional pathways

Among all the functional pathways identified in our analyses 2, 3, and 4, it is most interesting to examine those functions which showed significance in analysis 2 only, as such functions would not be confounded as identified our later results in analyses 3 and 4.

The pathways that were upregulated among case samples in analysis 2 that are not confounded by later results included connections to primary immunodeficiency (ko05340) and RNA degradation (ko03018), both of which have been researched in the cancer literature. Primary immunodeficiency has been found significantly enriched among gingivo-buccal oral cancer samples (Das et al., 2021). Moreover, Goodall and Wickramasinghe (2021) summarized mounting evidence on the alteration of RNAs as it contributes to cancer in a recent review.

Although Pyrimidine metabolism (PyM) was found to be upregulated among OSCC samples in the case vs. control analysis (Supplementary Figure 15), these results were confounded by the result that PyM was also significantly differentially regulated between the control samples only (Supplementary Figure 23). Recognizing the limitations of confounding, we note that PyM has been studied in the oncology literature. One recent review summarized recent conceptual advances on Pym and its dysfunction in relation to cancer progression (Wang et al., 2021). Our study found similar confounding results for PTT biosynthesis, which was shown to be downregulated among OSCC samples in the case vs. control analysis (Supplementary Figure 15) but also significantly differentially regulated among control samples only (Supplementary Figure 23). This confounding among results notwithstanding, we noted that PTT biosynthesis has previously been identified as increased among control samples in a study of OSCC (Al-Hebshi et al., 2017). Levels of phenylalanine and/or tryptophan have also been found to be decreased among case samples from patients across several studies of gastroesophageal cancer (Wiggins et al., 2015).

###  Toward establishing norms for data collection and analysis

While the data from our quantitative meta-analyses do suggest microbial relationships and functions which merit further study, the strongest evidence in these data indicates the need to standardize experimental designs and reporting of results in this growing field. The limitations to the meta-analytic work are numerous in the presence of such heterogeneity of data. In particular, aggregating data across multiple studies combines and compounds the biases that are known to be prevalent among contemporary analyses of oral microbiome data (Zaura et al., 2021). These sources of bias include variations in sample collection method, failure to control for known demographic confounders, differences in amplicon sequencing techniques, and inconsistent statistical methodology. Such limitations have been echoed within the wider context of head and neck squamous cell carcinoma (Metsäniitty et al., 2021). We recognize these limitations among the 11 studies examined in our present work, as well as the biases they may cause.

#### Sample collection method

At least three methods of collecting samples from patients were represented across our data sets: oral swabs, oral rinse/saliva, and tissue collection (Table 2). It has been documented that each different method for sample collection will yield a unique bacteriome (Gopinath et al., 2021; Zaura et al., 2021), and that saliva samples contain higher total DNA yield compared to oral swab samples (Wong et al., 2022). Whether it is possible to dissect out the critical differences between health and disease using samples of mixed type was debatable.

**Table 2:** Description of samples.

**First author**	**Sample collection method**	**Statistics reported**	**Sequencing tool**	**Region sequenced**
Pan	Swabs	Other	Wizard; ABI Prism 3100	Unknown
Guerrero-Preston	Tumor samples and salivary rinse	Relative abundance	Roche/454 GS Junior; 357F/926R primer set	V3–V5
Zhao	Swabs of various oral sites	Other	MiSeq	V4–V5
Mok	Swabs	Diversity index	EURx	V6–V9
Mukherjee	Tissue samples	Relative abundance	ITS1	V4
Bornigen	Oral rinse samples	Other	MiSeq	V4
Perera	Tissue samples	Diversity index and relative abundance	Gentra Puregene Tissue kit; 27FYm and 519R;MiSeq	V1–V3
Takahashi	Saliva	Relative abundance	MiSeq	V3–V4
Zhang	Bilateral buccal mucosal tissue scraping	Relative abundance	MiSeq;MOTHUR	V3–V4
Zhou	Tissue samples at various oral sites	Relative abundance	Illumina PE250 platform	V3–V4
Su	Swabs	Other	Qiamp; MiSeq; PICRUSt	V4

As a foil to this limitation, we were encouraged to see a recent example of a robust sample collection method described in Desai et al. (2022); these authors specified that their OSCC data was derived from only fresh-frozen primary tumors of tongue origin. We recommend that such specific criteria become the norm for studies in this field.

#### Demographic confounding

The case vs. case and control vs. control alpha diversity results (Supplementary Figures 18, 21) illustrated that the data from the Zhou study differed notably from the data in the Zhang and Zhao studies. This observation was also apparent in the case vs. case functional results (Supplementary Figure 20). It seemed plausible that the data from Zhang and Zhao were unusually similar, which may be explained by geography. Both the Zhang and Zhao data sets represented patients recruited at the Ninth People's Hospital in Shanghai, whereas the Zhou study was performed with participants in Qingdao, China. This difference in geography may imply that the participants from the Zhou data could have eaten a different diet and/or been exposed to different environments (e.g., climate) compared to participants from the other two studies. Moreover, each of the studies in our meta-analysis had different methods for taking into account participants' behavioral risk factors, such as tobacco and alcohol use. In the three studies represented in our aggregated data set, two authors supplied sample-specific information about patients' behavioral risk factors. For this reason, the extent to which possible confounding demographic factors added to or interacted with the existing differences in sample collection method remained unknown.

To mitigate demographic confounding, we suggest that others follow the example of Srivastava et al. (2022), wherein the authors narrowed their focus of their OSCC/microbiome investigation to patients who regularly used smokeless tobacco only.

#### Amplicon sequencing

As a result of the close connection between the field of microbiome research and the field of bioinformatics, changes in bioinformatics methods have a strong impact on the results of analysis in oral microbiome research. At least five different computing platforms for sequencing 16S rRNA data are represented among the studies in our present study (Table 2). Adding to this confounding is the variation in the regions targeted by these platforms. Although 16S rRNA sequencing is almost ubiquitous among the studies in our review, all regions V1–V9 are represented in the present data (Table 2). Such variation is likely to impact the results and conclusions drawn from downstream analyses (Kumar et al., 2011; Zaura et al., 2021). We hypothesize that differences in computing platforms and bioinformatic methods may be responsible for the discrepancies which we find between our analysis of data from individual studies and the analyses published by the original authors.

The shortcomings of amplicon sequencing are obvious in the sense that such sequencing only surveys the taxonomic composition of a bacteriome. Moreover, analysis pipelines for microbiome metagenomics data typically involve the use of a reference database for sequence alignment, taxonomic classification, and/or functional composition prediction. Among the five studies represented in our meta-analysis, we noted at least three reference data bases (KEGG, SILVA, GreenGenes) used in their respective analysis pipelines. Some authors have argued that the bias introduced by differences in analysis pipeline structure is the most significant challenge in metagenomic analyses (Escobar-Zepeda et al., 2018). Although computational methods such as PICRUSt and Tax4Fun (Aßhauer et al., 2015) can be used to infer functional potentials, it is not comparable to knowledge gained from more comprehensive omics data including whole genome meta-genomics and meta-transcriptomics.

#### Statistical methodology

Statistical methods and measures for quantifying diversity varied widely across the studies in our review. Relative abundance values and diversity indices such as the Inverse Simpson's index and Shannon's index were common, but by no means standard. From a data analytic perspective, such variation makes aggregating results difficult. We also noticed methods of normalization and rarefaction that have been criticized as ignorant of the compositional nature of microbiome data (Gloor et al., 2017).

Moreover, most studies did not provide high-quality, publicly accessible copies of their full data. Some authors chose to report only the most significant results, without providing a full table of results in the main text.

## Conclusions

In summary, our meta-analysis provided evidence of specific microbial genera, genes, and functional pathways having an association with OSCC status in oral cavity tissues. These potential biomarkers included an increased abundance of *Fusobacterium*, abundance of gene K06147 (ATP-binging cassette, subfamily B, bacterial), and upregulation/downregulation of many pathways such as ko03018 (RNA degradation).

The standardization of data collection, processing, and analysis techniques is an area of great need in the field of human oral microbiome study given the substantial decreases in sequencing costs and rapid increases in published studies. While it is both exciting and informative to study the human oral bacteriome and oral disease in meta- and mega- analyses, potentially powerful insights from future studies will be clouded by the confounding issues we have discussed due to the absence of standards in methodology, reporting, and data availability.

## Data availability statement

Our data set was aggregated from each of the studies by Takahashi et al. (2019), Zhang et al. (2020), Zhao et al. (2017), Zhou et al. (2020), and Perera et al. (2018). Each of those publications provided links to their respective data sets in publicly available repositories. This data can be found here: 1. Takahashi et al. (2019): NCBI BioProject accession number PRJNA525734. Link: https://www.ncbi.nlm.nih.gov/bioproject/PRJNA525734. 2. Zhang et al. (2020): NCBI BioProject accession number PRJNA533177. Link: https://www.ncbi.nlm.nih.gov/bioproject/PRJNA533177. 3. Zhao et al. (2017): NCBI BioProject accession number PRJNA362794. Link: https://www.ncbi.nlm.nih.gov/bioproject/PRJNA362794. 4. Zhou et al. (2020): NCBI BioProject accession number PRJNA597251. Link: https://www.ncbi.nlm.nih.gov/bioproject/PRJNA597251/. 5. Perera et al. (2018): NCBI BioProject accession number PRJNA415963. Link: https://www.ncbi.nlm.nih.gov/bioproject/PRJNA415963.

## Author contributions

TP implemented the non-parametric statistical tests (Wilcoxon and Kruskal-Wallis), synthesized the results, and wrote the first draft of the manuscript. MW performed all of the computations in LEfSe for the taxonomic, functional, and genetic data sets, created Venn diagrams, and collaborated with TP in drafting the manuscript. CC and CP completed the literature search. SD, SG, DD, and JB designed the meta-analysis comparisons and edited the manuscript. XX and EZ planned and designed the experiments, reviewed the manuscript, and mentored TP through the analysis process. All authors read and approved the final manuscript.

## Funding

JB, DD, SG, XX, and EZ were supported by University of Iowa Strategic Initiatives Fund: P3 Program in Support of Strategic Priorities. EZ was supported by University of Iowa College of Dentistry Seed Grant.

## Conflict of interest

The authors declare that the research was conducted in the absence of any commercial or financial relationships that could be construed as a potential conflict of interest.

## Publisher's note

All claims expressed in this article are solely those of the authors and do not necessarily represent those of their affiliated organizations, or those of the publisher, the editors and the reviewers. Any product that may be evaluated in this article, or claim that may be made by its manufacturer, is not guaranteed or endorsed by the publisher.

## Footnotes

^1^The study by Zhou et al. (2020) had a single oropharynx sample, which we excluded from our meta-analysis data.

^2^The investigators of these projects reported age differently, with some reporting means and others reporting medians.

^3^Some data named genera which were not detected in any samples among the aggregated data set.

## Abbreviations

ASV: amplicon sequencing variant
GreenGenes: An online database comprised of 16S rRNA genes organized formats for use in pipelines
HOMD: human oral microbiome database online resource)
KEGG: Kyoto encyclopedia of genes and genomes (an online resource)
LEfSe, Linear discriminant analysis effect size (from Segata et al.: 2011)
OSCC: oral squamous cell carcinoma
PTT (biosynthesis), phenylalanine, tyrosine: and tryptophan
PyM: pyrimidine metabolism
SILVA: a comprehensive online resource for quality checked and aligned ribosomal RNA sequence data.
