# Supplementary material for: Systematic review and meta-analysis of oral squamous cell carcinoma associated oral microbiome
Source: Front Microbiol. 2022 Oct 20;13:968304. doi: 10.3389/fmicb.2022.968304 (PMC9632422; doi:10.3389/fmicb.2022.968304)
Supplement: Supplementary file 3 [file Data_Sheet_1.pdf]

Supplementary Material

Details for analysis 1 (case v. control)

The first component of our taxonomic meta-analysis re-examined the results of the five original studies using our data processing workflow and our analytic methods. Below, we summarize the findings from this re-examination and compare our findings to those published in the original studies.

Regarding the limitations of this re-examination, we noted the following:

1. As noted in the main text, the data from the studies by Zhou et al. (2020) and Perera et al. (2018) represented tissue samples, while the three of the other studies collected samples via saliva samples or swabs (Takahashi et al. (2019), Zhang et al. (2020), Zhao et al. (2017)). The structure of our analysis 1 results reflects this important distinction.
2. Perera and colleagues used oral fibroepithelial polyp (FEP) tissues as a control, rather than histologically normal tissues.
3. Among the three studies of paired design, only one author (Zhao) indicated a 1-to-1 mapping of which samples were paired. The other two studies with an intra-subject control design did not indicate which samples came from the same subject, so implementing paired tests was not possible.

   Throughout our supplementary material, we used labels A-E to abbreviate the five studies Zhang et al. (2020), Zhao et al. (2017), Zhou et al. (2020), Takahashi et al. (2019), and Perera et al. (2018), respectively.

Alpha and beta diversity

All five studies reported the Shannon diversity index as a measure of alpha diversity. Studies C, D, and E reported no evidence for a difference in alpha diversity, while studies A and B each reported significantly higher alpha diversity in OSCC samples. We assessed alpha diversity for the samples from each of these studies using Wilcoxon tests, and found results discordant with those reported by studies A and C (Figure S1).

We found that some of our calculations of Shannon's index differed from the original studies. For example, Study E reported Shannon's index values of 3.3 and 3.6 for the OSCC and FEP groups, respectively. However, our analysis calculated these values to be 1.85 and 1.75 [1]. Such differences may be attributable to the impact of preprocessing methods on generating data tables for taxonomic data. The alpha diversity results also illustrated evidence of the distinct compositions of the data in studies C and E, which is likely attributable to these authors using tissue samples (as opposed to saliva or oral swab samples).

Regarding beta (between-sample) diversity, Studies A, C, D, and E each reported evidence of dissimilarity between the microbial community structures of the OSCC and control groups. Study B reported mixed results, with significance varying according to the method used to assess beta-diversity.

[1] both the original analysis and our analysis found that this difference in alpha diversity was not statistically significant.

These five studies used different methods for examining beta diversity, making it difficult to draw comparisons between the original results and our replicated analyses. For studies A-C (those studies which were represented in our merged data set for our case v. case and control v. control analyses), we chose the weighted UniFrac measure as our method of measuring beta diversity, since this method takes phylogenetic information into account (Lozupone et al. (2011)). Our plot of the weighted UniFrac measurements illustrated that among the samples from the three studies with intra-subject control designs, clustering patterns between case/control groups were subtle, whereas clustering patterns between data sets were obvious (Figure S2).

Taxonomic and functional results from OSCC case samples

Among the three data sets representing saliva/oral swab samples (studies A, B, and D), we found that the genera Fusobacterium, Peptostreptococcus, Capnocytophaga, Alloprevotella, Parvimonas, and Johnsonella were consistently more abundant in case samples (Figures S3, S4, S6). From the functional results, a pathway associated with plant-pathogen interaction (ko04626) was significantly more abundant in OSCC samples from these saliva/oral swab data sets (Figures S9, S11).

The Study C data was concordant with the other studies' results in that Peptostreptococcus and Parvimonas were found more abundant in OSCC samples; however, Fusobacterium did not show significance in either the case or control groups (Figure S5). A pathway associated with human papillomavirus infection (ko05165) was more abundant in OSCC samples from this tissue sample data set; we noticed that this pathway was not found significant in any of the saliva/oral swab data sets (Figure S10).

Our analysis of Study E identified Fusobacterium, Capnocytophaga, and Parvimonas as significantly differentially abundant in OSCC deep tissue samples (Figure S7) at a notable magnitude (LDA score < -3). Notably, the original publication did not identify Fusobacterium and Parvimonas as having the same magnitude of significant differential abundance in the OSCC samples. This difference in magnitude may be attributable to the impact of changes in analysis pipeline, as a major update to the QIIME platform has been released since the publication of Perera's work (Bolyen et al. (2019)). The predicted functional pathways found to be significantly differentially more abundant in the OSCC samples in Study E were not identified in the other studies (Figure S12). We hypothesized that the uniqueness of the functional results in Study E may be attributable to the use of FEP tissues samples as controls.

Taxonomic and functional results from control samples

Among the three data sets representing saliva/oral swab samples (Studies A, B, and D), there was no genus that was consistently more abundant in control samples. In the tissue sample data set from Study C, there were several genera that appeared as significantly more abundant in case samples; many of these were not named in any of the results from the saliva/oral swab data sets, including Allorhizobium, Photobacterium, and Sphingomonas. The only functional pathway found to be increased in control samples in at least three studies was inositol phosphate metabolism.

There was some discordance in the direction of the significance between the saliva/oral swab data sets and the tissue sample data set. In the taxonomic results, genus Streptococcus was identified as more abundant in the case samples from the Study C data set, whereas this genus was more abundant in the control samples in the data sets from studies A and B (see S16). From the functional results, pathways associated with ribosome biogenesis (ko03008) and phenylalanine, tyrosine, and

tryptophan (PTT) biosynthesis (ko00400) were more abundant in the case samples from the Study C data set, but more abundant in the control samples from the data sets in Studies A-B (see S17).

Remarks on choice of merging data sets

We began our exploration by categorizing our five data sets (A-E) according to the type of control sample used in the design. Studies A, B, and C all used intra-control designs, so we merged the data sets from these studies into a single data set. This merged data has served as the basis for most of our analysis.

Although studies D and E both used inter-control designs, the authors of these studies chose different definitions of "control." In Study D (Takahashi et al. (2019)), the authors defined their criteria for control samples as saliva samples from "individuals without any diagnosed mucosal diseases and other cancers." Alternatively, the authors of Study E (Perera et al. (2018)) defined their criteria for control samples as deep tissue samples with "FEP from the same anatomic sites" as those of the OSCC samples. Since the definitions of control samples differed notably between these two studies, we chose not to pursue merging the data from studies D and E in the present work.

Details for analysis 2 (merged case v. merged control)

In this analysis, we combined the samples from the three studies with an intra-subject control design to form a merged case group of 113 samples and a control group of 113 samples. All of these samples came from the oral cavity (including the tongue). [2].

Wilcoxon tests compared the log-transformed ratio of median proportions as a measure of relative abundance. These tests identified 20 genera as significantly differentially abundant between the merged case and control groups, with Fusobacterium being among the most significant results (Figure S13). Note that in Figure S13, the log (base 2) transformed ratio represents a measure of effect size. A ratio above 0 indicates that a genus was more abundant in the OSCC samples, whereas a ratio below 0 indicates that genus was more abundant in control samples [3] [4].

Regarding alpha diversity in the merged data, the Shannon index showed no evidence of a difference between case and control groups (Figure S14).

Using LEfSe, we compared the functional pathways between samples from the merged case and merged control groups (Figure S15).

The genera found to be significant in analyses 1 and 2 were summarized in a Venn diagram (Figure S16). Three genera showed significance in each of the three studies and in the merged analysis; these were Streptococcus, Parvimonas, and Peptostreptococcus. As described in the introduction section of the main text, the direction of significance (i.e. over-representation in case or control samples) of Streptococcus was inconsistent among these studies. However, all three of the data sets with intra-subject control samples found both Parvimonas and Peptostreptococcus to have a higher abundance in the OSCC samples. This echoed the findings of recent studies of colorectal cancer, which reported that Parvimonas micra, Peptostreptococcus stomatis, and Fusobacterium nucleatum were over-represented in the gut microbiomes of colorectal cancer patients (Osman et al.

[2] NB: The data from Zhou et al. included one patient with case and control samples from the oropharynx. We excluded data representing these samples from our analysis.

[3] results are in ascending order by FDR-adjusted p-value.

[4] Where genera had no counts in the control group, the ratio is incalculable and labeled as 'Inf.' Ratios near zero indicate nearly even abundance between OSCC and control groups.

(2021), Löwenmark et al. (2022)). Taken together, these results suggest that a combination of species from Parvimonas, Peptostreptococcus, and Fusobacterium merit further study as having a possible association with the etiology of multiple cancers.

The functional pathways found to be significant in analyses 1 and 2 were summarized in a Venn diagram (Figure S17). The summarized results show that the merged analysis (Analysis 2) does not generate new significant pathways, instead it circles shared significant pathways identified from the data sets of three independent studies. As shown in the center of the figure, two functional pathways were found to be significant in all three studies individually and in the merged analysis: Inositol phosphate metabolism (ko00562) and PTT biosynthesis (ko00400). These were both downregulated in case samples.

Members of the inositol phosphate metabolism pathway regulate cell proliferation, migration and phosphatidylinositol-3-kinase (PI3K)/Akt signaling. One recent study reported that dysregulated inositol phosphate metabolism pathway in humans was significantly associated with risk of a number of cancers (Tan et al. (2015)). Another study indicated that a bacterial homolog of a eukaryotic inositol phosphate signaling enzyme mediates cross-kingdom dialog in the mammalian gut (Stentz et al. (2014)), and a third study reviewed the roles inositol phosphates in energy metabolism (Chatree et al. (2020)). The finding from our meta-analysis of oral microbiome might give a hint about bacteria-host interaction in oral cancer through inositol phosphate metabolites.

In addition, PTT is a natural-product antibiotic and potent herbicide produced by Streptomyces hygroscopicus ATCC 21705 and Streptomyces viridochromogenes DSM 40736 (Metcalf and Van Der Donk (2009), Blodgett et al. (2007)). Moreover, it has recently been posited that serum amino acids have a prognostic role in head and neck cancer (Cadoni et al. (2020)); however, its role in anti-cancer has not yet been well documented and merits future investigation.

Details for analysis 3

Analysis 3 compared the case (OSCC) samples between the Zhang (n = 50), Zhao (n = 40), and Zhou (n = 23) studies. Kruskal-Wallis tests were used to examine the differences in alpha diversity between these three data sets. These tests provided evidence of significant differences in both the Shannon's index (Figure S18). Boxplots representing these alpha diversity metrics reaffirmed that the Zhou data set differed notably from the rest of the data.

LEfSe plots comparing the taxonomic composition of the case samples at the genus level are shown in Figure S19. LEfSe identified over 30 genera that were significantly differentiated among Zhang, Zhao, and Zhou studies. This result indicated heterogeneity among case samples and confirmed the presence of confounding factors in the results. The results from the Kruskal-Wallis tests of relative abundance found 86 significant differences at an FDR level of 0.05 (Supplemental data set 1), which echoed this evidence of confounding.

LEfSe comparisons of the functional pathways in the case samples between the Zhang, Zhao, and Zhou studies are shown in Figure S20. As with the comparison of taxonomic data, there was evidence of heterogeneity. Here again, the data showed evidence of the Zhou data being notably different.

Details for analysis 4

Using Kruskal-Wallis tests, we compared alpha diversity among the control samples from the Zhang, Zhao, and Zhou studies. The data from the control samples did not show evidence of a difference in alpha diversity (Figure S21)

Our LEfSe analysis of the control samples showed evidence of significant differential abundance for more than 30 genera (Figure S22), and the Kruskal-Wallis tests identified significant differences in relative abundance for 114 genera (Supplemental data set 2). This further illustrated the notable impact of confounding variables in these data.

LEfSe analyses of the functional pathways among the control samples also showed significant differences (Figure S23). This provided further evidence of confounding.

1 NOMENCLATURE

Additional notes on nomenclature and abbreviations in this work are as follows:

- FEP: Fibroepithelial polyp
- KEGG: Kyoto encyclopedia of genes and genomes (an online resource)
- LEfSe: linear discriminant analysis effect size (from Segata et al. (2011))
- QIIME: Quantitative Insights into Microbial Ecology (from Bolyen et al. (2019)).
- Study A: Zhang et al. (2020)
- Study B: Zhao et al. (2017)
- Study C: Zhou et al. (2020)
- Study D: Takahashi et al. (2019)
- Study E: Perera et al. (2018)
- OSCC: oral squamous cell carcinoma
- PyM: pyrimidine metabolism
- PTT (biosynthesis): phenylalanine, tyrosine, and tryptophan

REFERENCES

Blodgett, J. A., Thomas, P. M., Li, G., Velasquez, J. E., Van Der Donk, W. A., Kelleher, N. L., et al. (2007). Unusual transformations in the biosynthesis of the antibiotic phosphinothricin tripeptide. Nature chemical biology 3, 480–485

Bolyen, E., Rideout, J. R., Dillon, M. R., Bokulich, N. A., Abnet, C. C., Al-Ghalith, G. A., et al. (2019). Reproducible, interactive, scalable and extensible microbiome data science using qiime 2. Nature Biotechnology 37, 852–857. doi:10.1038/s41587-019-0209-9

Cadoni, G., Giraldi, L., Chiarla, C., Gervasoni, J., Persichilli, S., Primiano, A., et al. (2020). Prognostic role of serum amino acids in head and neck cancer. Disease Markers 2020

Chatree, S., Thongmaen, N., Tantivejkul, K., Sitticharoon, C., and Vucenik, I. (2020). Role of inositols and inositol phosphates in energy metabolism. Molecules 25, 5079

Löwenmark, T., Li, X., Löfgren-Burström, A., Zingmark, C., Ling, A., Kellgren, T. G., et al. (2022). Parvimonas micra is associated with tumour immune profiles in molecular subtypes of colorectal cancer. Cancer Immunology, Immunotherapy , 1–11

Lozupone, C., Lladser, M. E., Knights, D., Stombaugh, J., and Knight, R. (2011). Unifrac: an effective distance metric for microbial community comparison. The ISME journal 5, 169–172

Metcalf, W. W. and Van Der Donk, W. A. (2009). Biosynthesis of phosphonic and phosphinic acid natural products. Annual review of biochemistry 78, 65–94

Osman, M. A., Neoh, H.-m., Ab Mutalib, N.-S., Chin, S.-F., Mazlan, L., Raja Ali, R. A., et al. (2021). Parvimonas micra, peptostreptococcus stomatis, fusobacterium nucleatum and akkermansia muciniphila as a four-bacteria biomarker panel of colorectal cancer. Scientific reports 11, 1–12

Perera, M., Al-Hebshi, N., Perera, I., Ipe, D., Ulett, G., Speicher, D., et al. (2018). Inflammatory bacteriome and oral squamous cell carcinoma. Journal of dental research 97, 725–732

Segata, N., Izard, J., Waldron, L., Gevers, D., Miropolsky, L., Garrett, W. S., et al. (2011). Metagenomic biomarker discovery and explanation. Genome Biology 12, R60. doi:10.1186/gb-2011-12-6-r60

Stentz, R., Osborne, S., Horn, N., Li, A. W., Hautefort, I., Bongaerts, R., et al. (2014). A bacterial homolog of a eukaryotic inositol phosphate signaling enzyme mediates cross-kingdom dialog in the mammalian gut. Cell reports 6, 646–656

Takahashi, Y., Park, J., Hosomi, K., Yamada, T., Kobayashi, A., Yamaguchi, Y., et al. (2019). Analysis of oral microbiota in Japanese oral cancer patients using 16S rRNA sequencing. Journal of Oral Biosciences 61, 120–128. doi:https://doi.org/10.1016/j.job.2019.03.003

Tan, J., Yu, C.-Y., Wang, Z.-H., Chen, H.-Y., Guan, J., Chen, Y.-X., et al. (2015). Genetic variants in the inositol phosphate metabolism pathway and risk of different types of cancer. Scientific reports 5, 1–8

Zhang, L., Liu, Y., Zheng, H. J., and Zhang, C. P. (2020). The Oral Microbiota May Have Influence on Oral Cancer 9, 476

Zhao, H., Chu, M., Huang, Z., Yang, X., Ran, S., Hu, B., et al. (2017). Variations in oral microbiota associated with oral cancer. Scientific Reports 7, 11773. doi:10.1038/s41598-017-11779-9

Zhou, J., Wang, L., Yuan, R., Yu, X., Chen, Z., Yang, F., et al. (2020). Signatures of mucosal microbiome in oral squamous cell carcinoma identified using a random forest model. Cancer Management and Research 12, 5353

2 FIGURE CAPTIONS

1. Figure S1: Comparing alpha diversity. These boxplots represent Shannon index values in case and control samples within each study, e.g. case samples in study A v. control samples in study A. Comparisons were done with Wilcoxon rank sum tests; corresponding p-values are shown above.
2. Figure S2: Emperor plot of weighted UniFrac measurements in studies A-C. This plot illustrates the first three ordinates of a PCoA, showing that clustering is most apparent between studies rather than between OSCC/control status. The plot was created using QIIME2 (Bolyen et al. (2019)).
3. Figure S3: Taxonomic LEfSe results - Zhang et al.
4. Figure S4: Taxonomic LEfSe results - Zhao et al.
5. Figure S5: Taxonomic LEfSe results - Zhou et al.
6. Figure S6: Taxonomic LEfSe results - Takahashi et al.

7. Figure S7: Taxonomic LEfSe results - Perera et al.
8. Figure S8: Functional LEfSe results - Zhang et al.
9. Figure S9: Functional LEfSe results - Zhao et al.
10. Figure S10: Functional LEfSe results - Zhou et al.
11. Figure S11: Functional LEfSe results - Takahashi et al.
12. Figure S12: Functional LEfSe results - Perera et al.
13. Figure S13: Wilcoxon test results (case v. control) - Studies A-C. We noted that several of these results overlapped with the case v. control LEfSe plot of taxonomic data (Figure 3 in the main text), including Fusobacterium and Peptostreptococcus being significantly more abundant in case samples.
14. Figure S14: Comparison of Shannon's index (case v. control) - Studies A-C
15. Figure S15: Functional LEfSe (case v. control) - Studies A-C. K0 numbers for the functional pathways are listed alongside the pathway names.
16. Figure S16: Summary of taxonomic LEfSe results (Analyses 1 and 2) - Studies A-C. This Venn diagram illustrates the overlap of the number of genera found to be significantly differentially abundant in case v. control comparisons within the individual data sets from Studies A-C and/or the merged data set. We noted that three genera showed significance in each of the three studies and in the merged analysis; these were Streptococcus (enriched in controls (A-B) and in OSCC (C)), Parvimonas (enriched in OSCC), and Peptostreptococcus (enriched in OSCC).
17. Figure S17: Summary of functional LEfSe results (Analyses 1 and 2) - Studies A-C. This Venn diagram illustrates the overlap of the number of functional pathways found to be significantly differentially abundant in case v. control comparisons within the individual data sets from Studies A-C and/or the merged data set. We noted that 2 pathways showed significance in each of the three studies and in the merged analysis: inositol phosphate metabolism (ko00562 - downregulated in OSCC samples) and PTT biosynthesis (ko00400 - downregulated in OSCC samples in studies A-B, but upregulated in OSCC samples in study C).
18. Figure S18: Shannon index comparison (case v. case) - Studies A-C. These boxplots illustrate that alpha diversity differs significantly among the case samples from Studies A-C. Significance was assessed using the Kruskal-Wallis test (a non-parametric analog to ANOVA).
19. Figure S19: Taxonomic LEfSe (case v. case) - Studies A-C. This plot illustrates that there are significant differences in abundance at the genus level in the case samples from Studies A-C. We presented these differences as evidence of confounding factors in the case v. control analysis.
20. Figure S20: Functional LEfSe (case v. case) - Studies A-C. This plot illustrates that there are significant differences in abundance in the functional pathways in the case samples from Studies A-C. We noted that the case samples from Study C differed notably from the other case samples in our merged data set.
21. Figure S21: Shannon index comparison (control v. control) - Studies A-C. These boxplots show no evidence of a difference in alpha diversity between the control samples of Studies A-C. Significance was assessed using the Kruskal-Wallis test (a non-parametric analog to ANOVA).
22. Figure S22: Taxonomic LEfSe (control v. control) - Studies A-C. This plot illustrates that there are significant differences in abundance at the genus level in the control samples from Studies

A-C. We presented these differences as evidence of confounding factors in the case v. control analysis.

23. Figure S23: Functional LEfSe (control v. control) - Studies A-C. This plot illustrates that there are significant differences in the functional pathways in the control samples from Studies A-C.

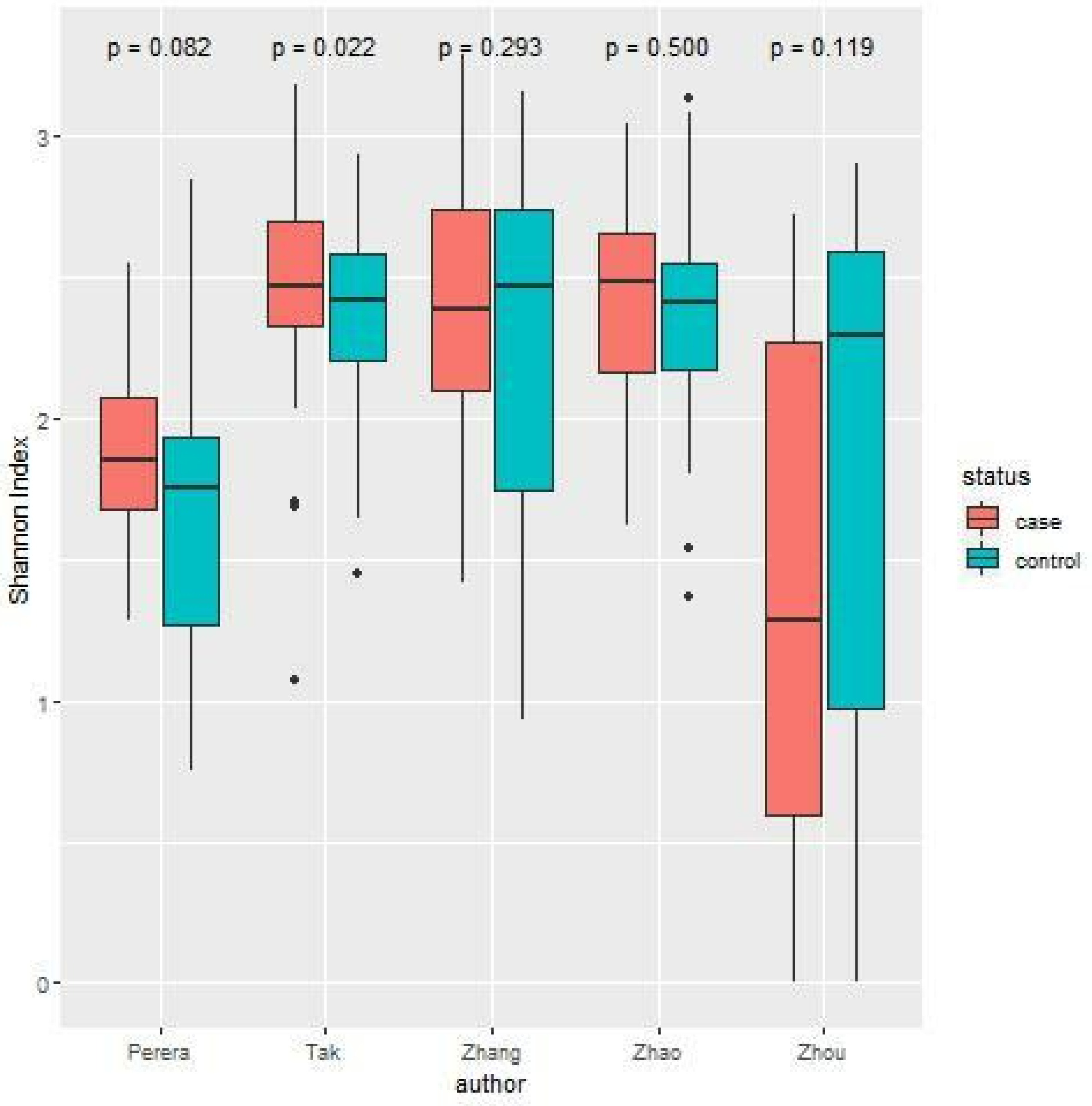

Figure S1. Comparing Alpha Diversity (Shannon diversity index, studies A-E)

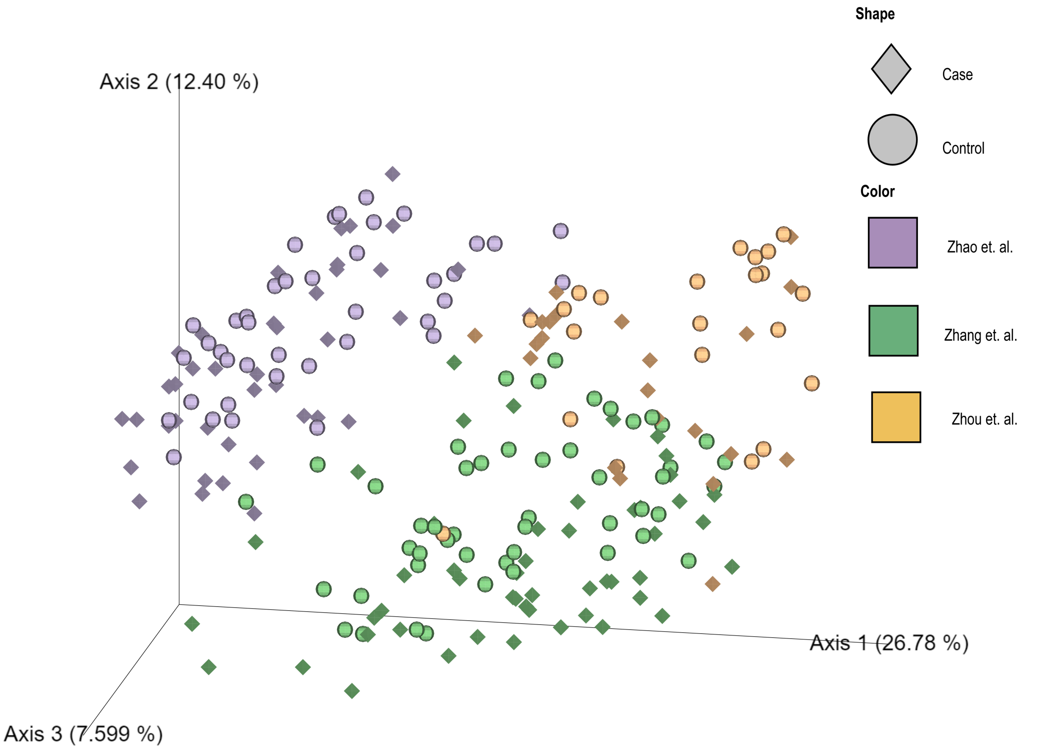

Figure S2. Emperor plot of weighted UniFrac, studies A-C

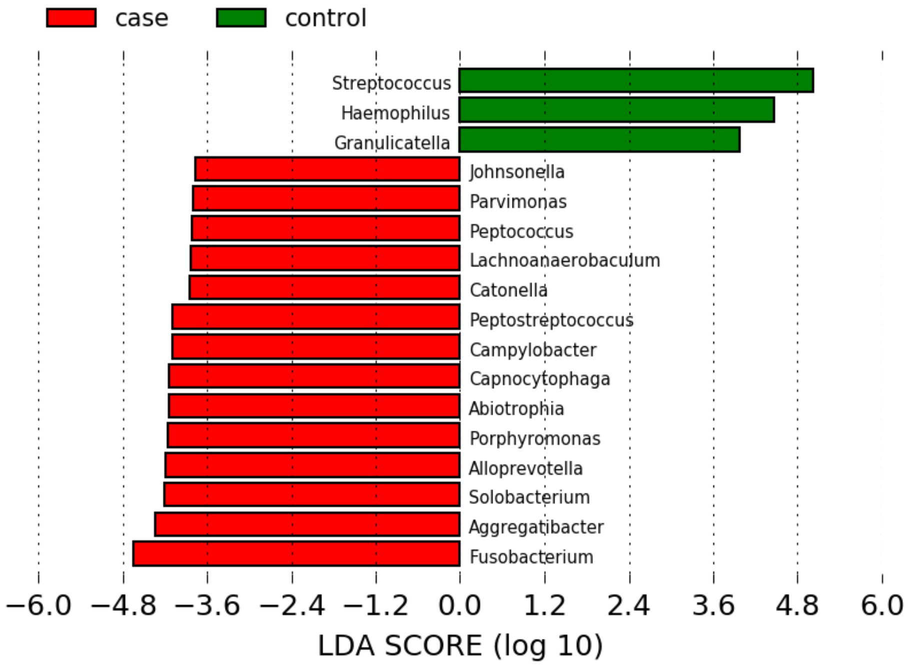

Figure S3. Taxonomic LEfSe - Zhang et al.

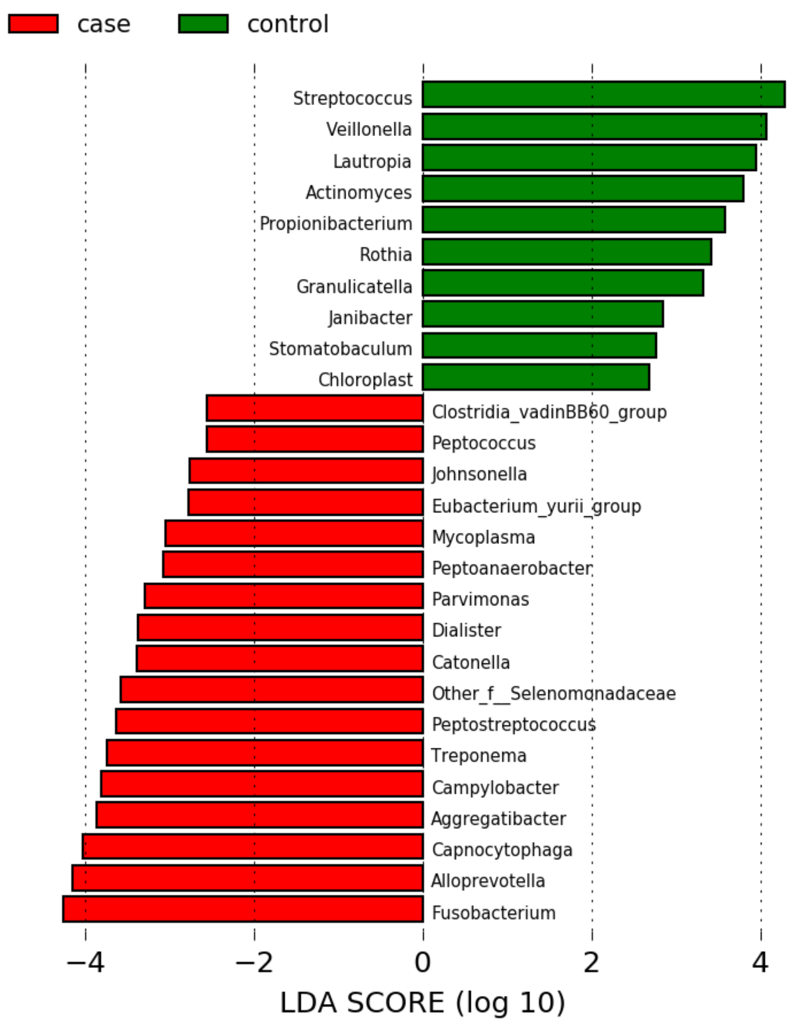

Figure S4. Taxonomic LEfSe - Zhao et al.

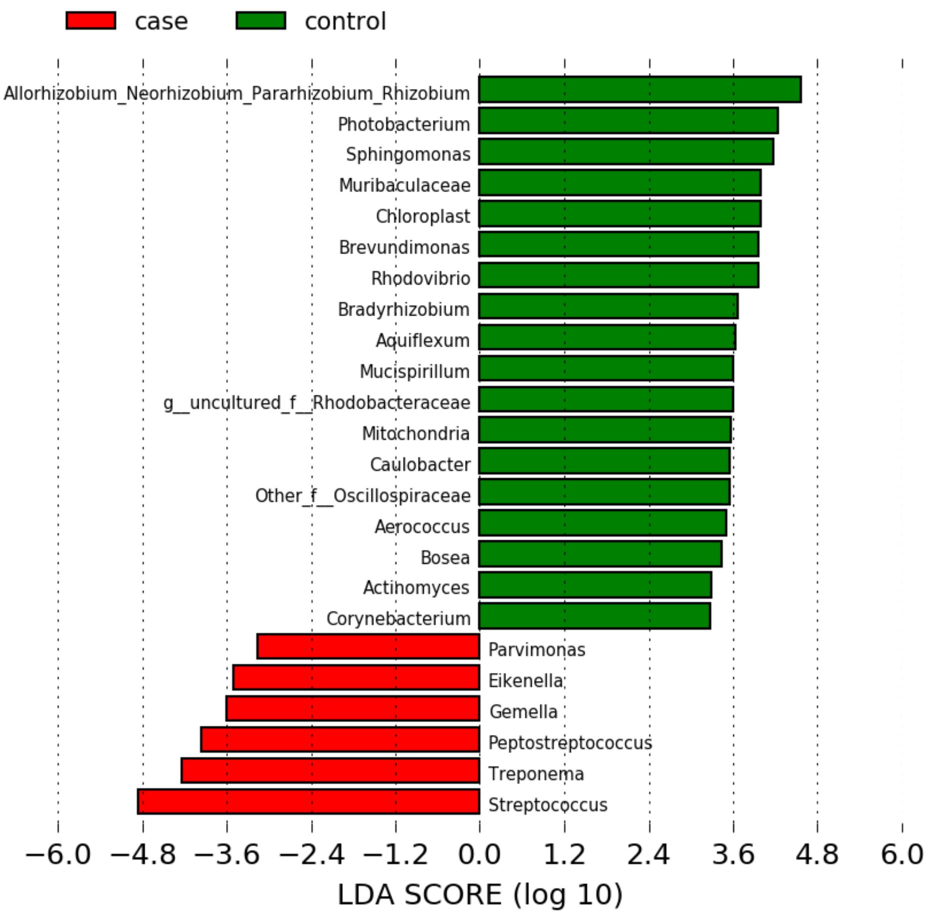

Figure S5. Taxonomic LEfSe - Zhou et al.

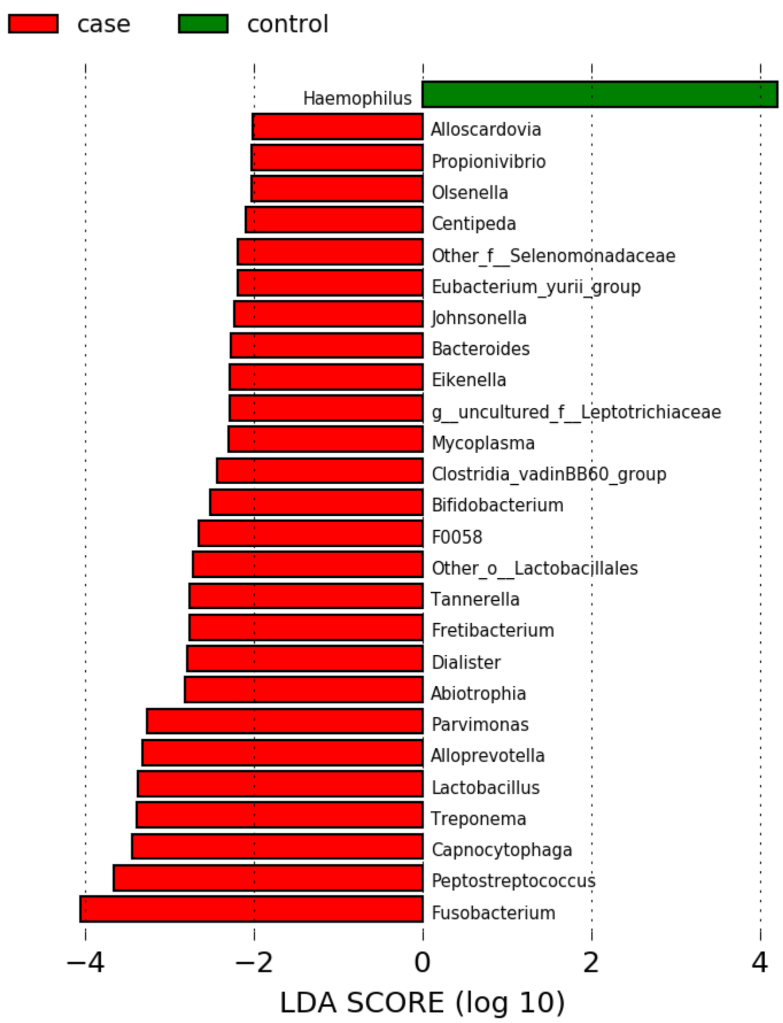

Figure S6. Taxonomic LEfSe - Takahashi et al.

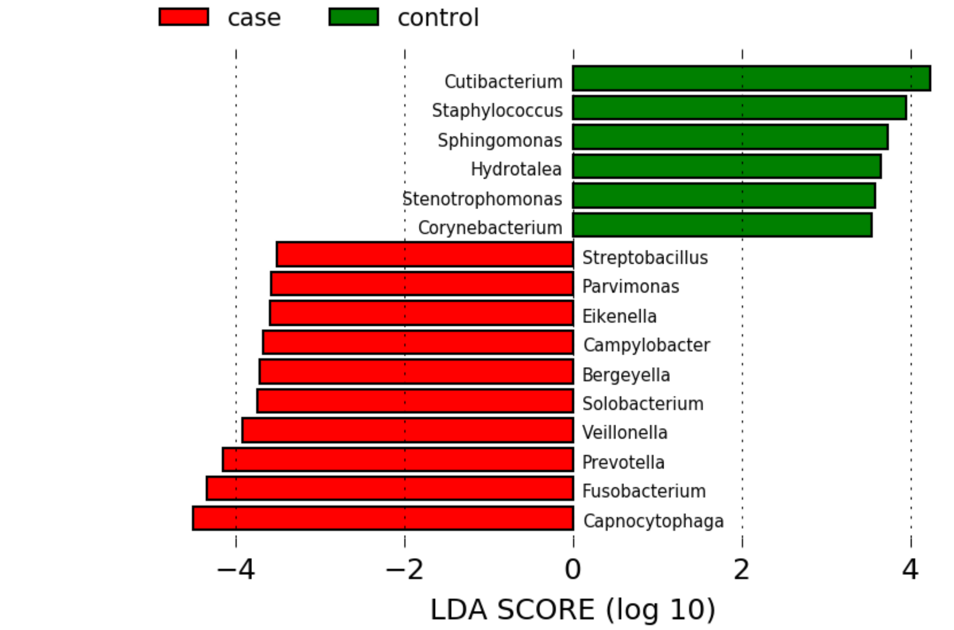

Figure S7. Taxonomic LEfSe - Perera et al.

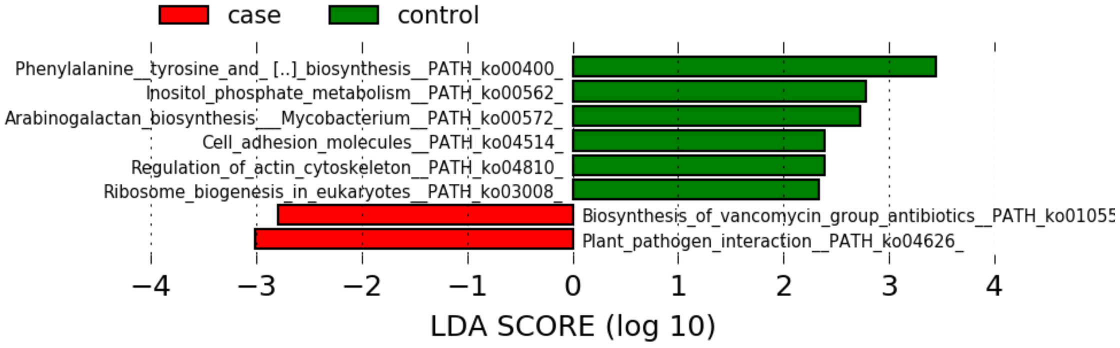

Figure S8. Functional LEfSe - Zhang et al.

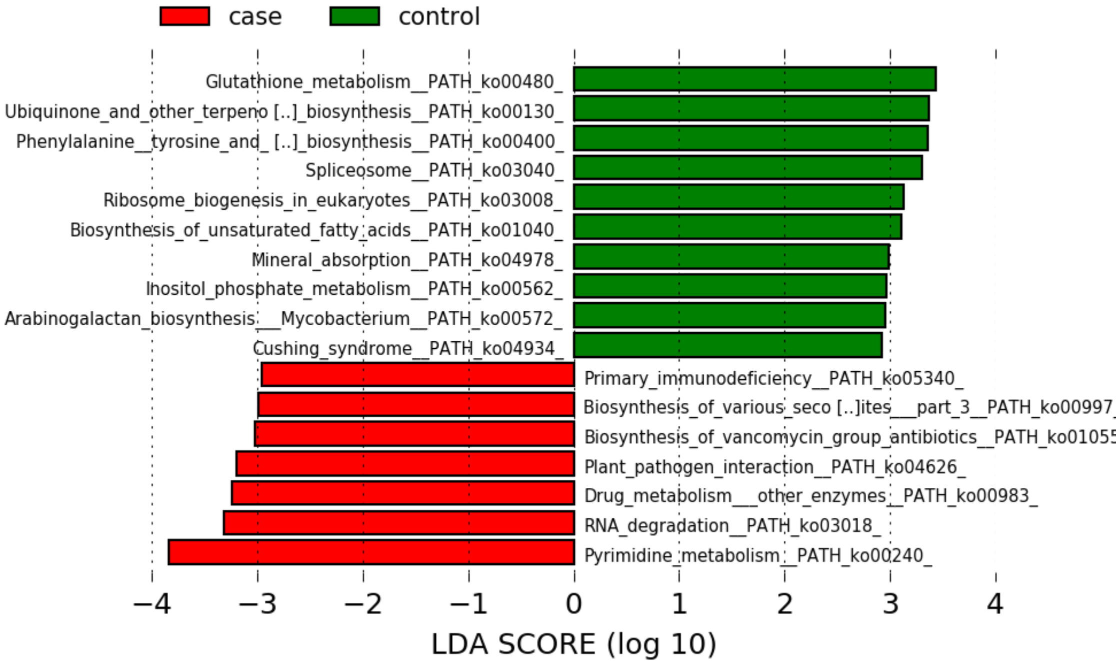

Figure S9. Functional LEfSe - Zhao et al.

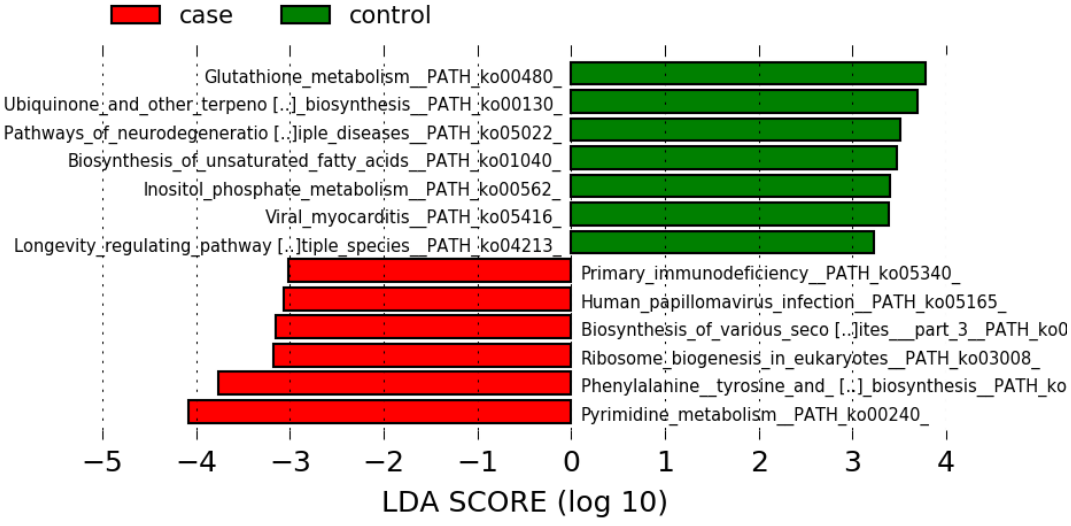

Figure S10. Functional LEfSe - Zhou et al.

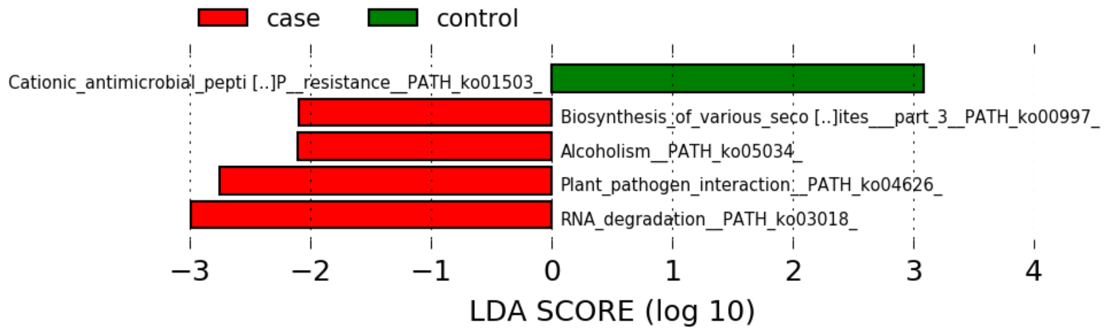

Figure S11. Functional LEfSe - Takahashi et al.

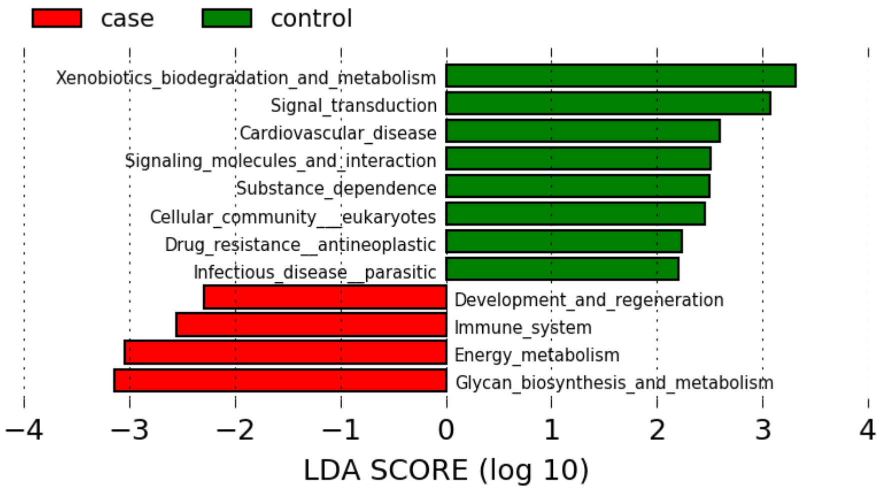

Figure S12. Functional LEfSe - Perera et al.

tax_name	wilcox_p_value	log2_median_ratio
Fusobacterium	0.0001658798	1.7235647
Peptostreptococcus	0.0002501127	3.2180078
Rothia	0.0021202836	-3.7906276
Actinomyces	0.0040148653	-3.6649841
Granulicatella	0.0040148653	-1.2873604
Parvimonas	0.0040148653	Inf
Peptococcus	0.0111831489	Inf
Campylobacter	0.0127454689	1.1789946
Brevundimonas	0.0185668422	0.0000000
Chloroplast	0.0193938017	0.0000000
Treponema	0.0193938017	1.8302120
Alloprevotella	0.0195211421	1.1744172
Streptococcus	0.0334568072	-0.7989726
[Eubacterium]_yurii_group	0.0334568072	0.0000000
Mitochondria	0.0334568072	0.0000000
Veillonella	0.0398661108	-1.7605308
Peptoanaerobacter	0.0398962835	0.0000000
Johnsonella	0.0458553275	0.0000000
Catonella	0.0478724775	2.1008999
Stomatobaculum	0.0486257714	0.0000000

Figure S13. Wilcoxon test results (case v. control) - Studies A-C

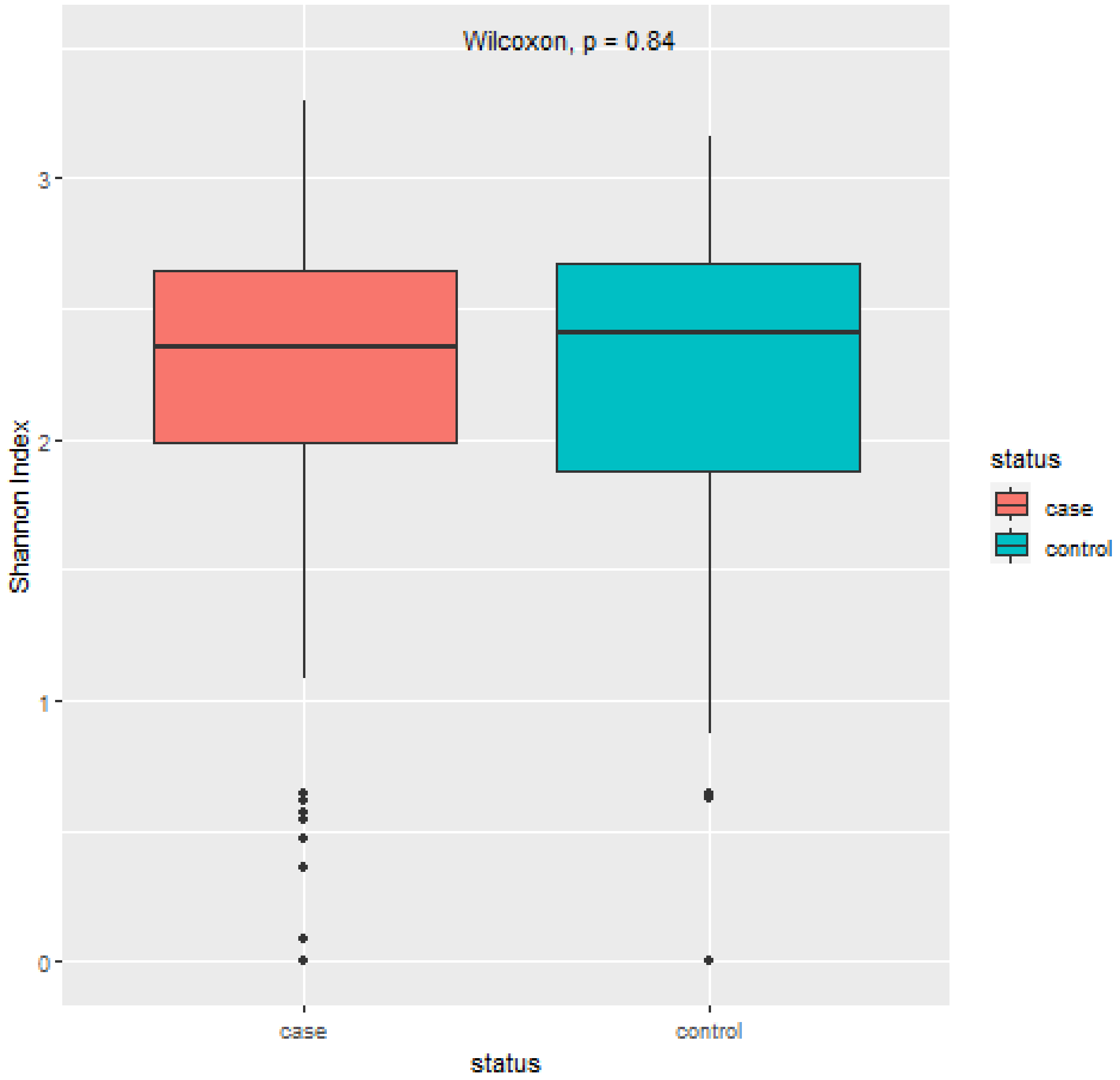

Figure S14. Comparison of Shannon's index (case v. control) - Studies A-C

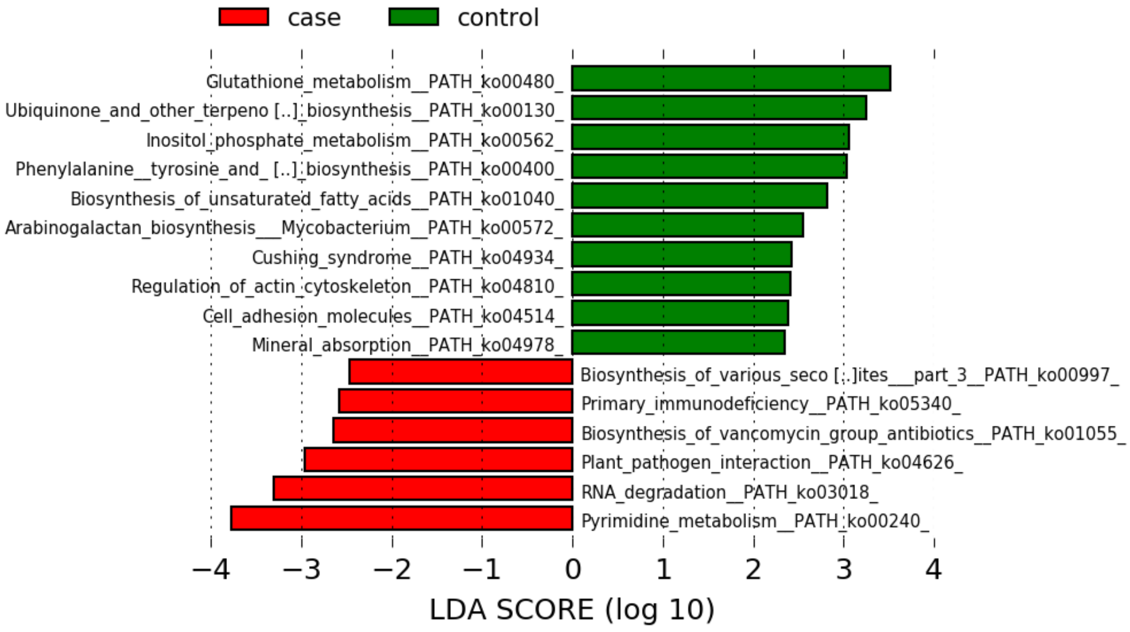

Figure S15. Functional LEfSe (case v. control) - Studies A-C

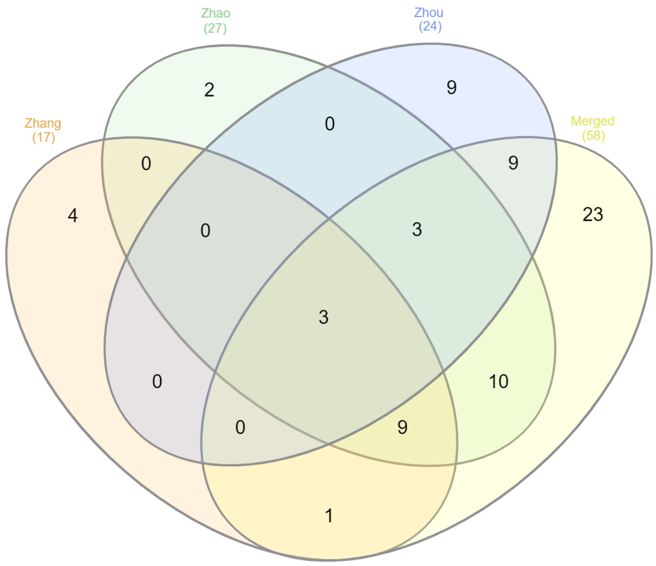

Figure S16. Summary of Taxonomic LEfSe Results (Analyses 1 and 2) - Studies A-C

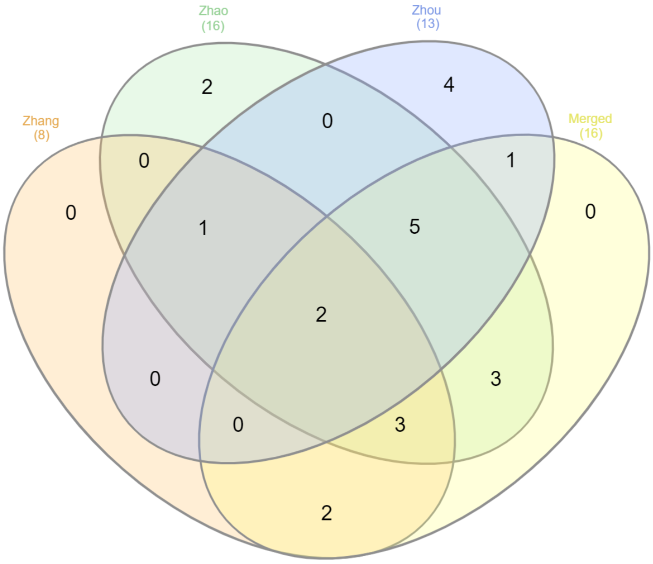

Figure S17. Summary of Functional LEfSe Results (Analyses 1 and 2) - Studies A-C

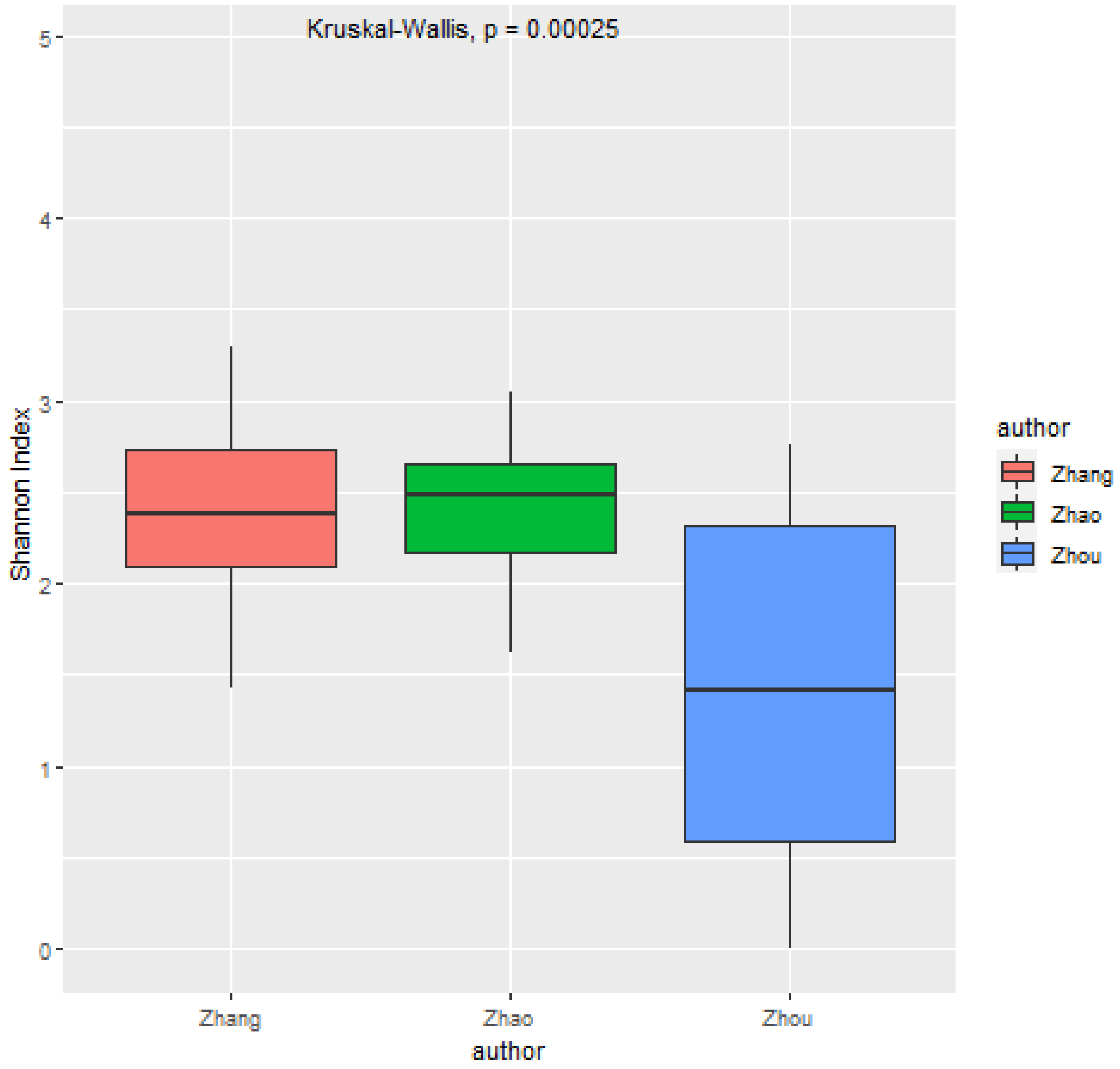

Figure S18. Shannon index comparison (case v. case) - Studies A-C

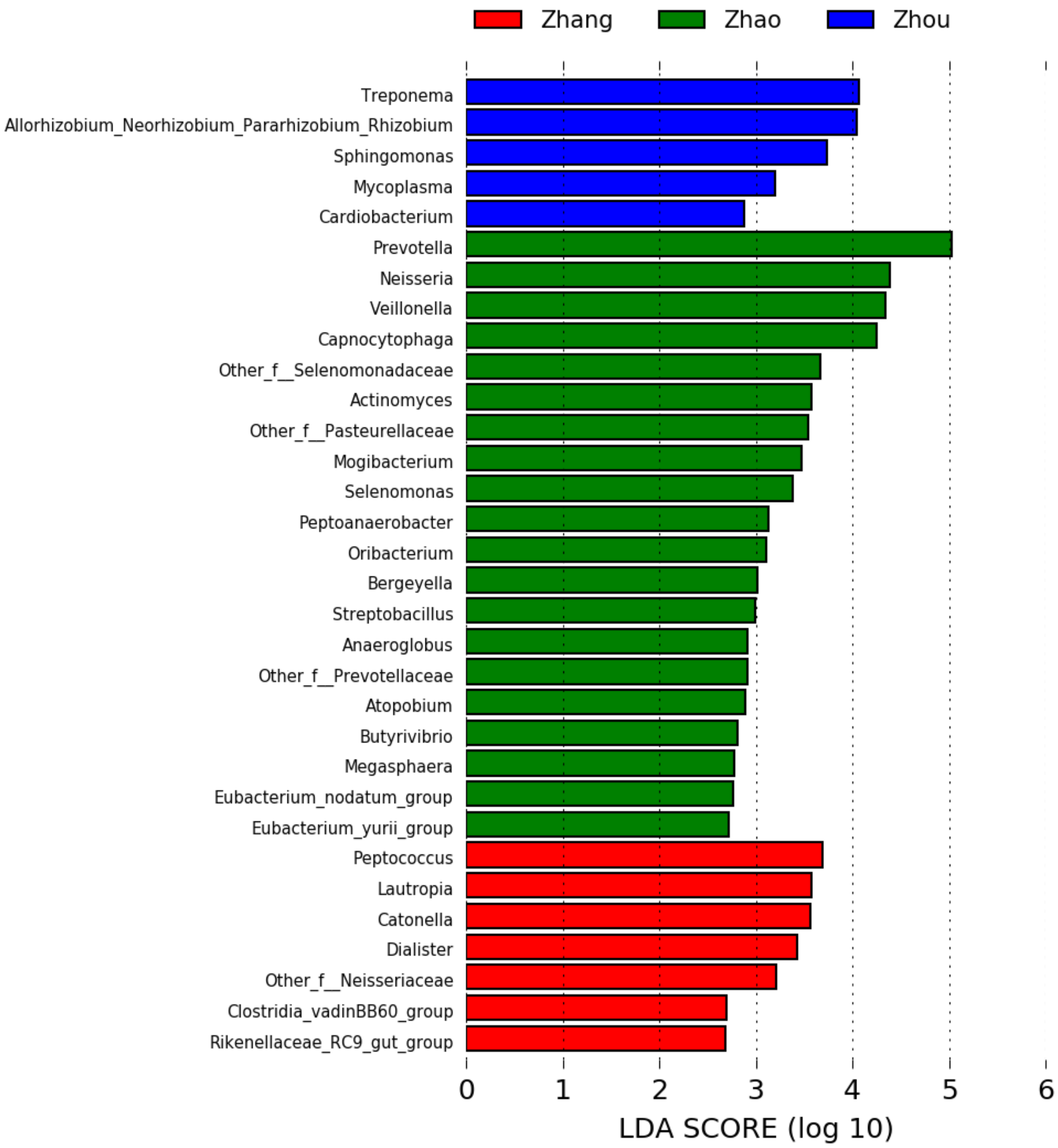

Figure S19. Taxonomic LEfSe (case v. case) - Studies A-C

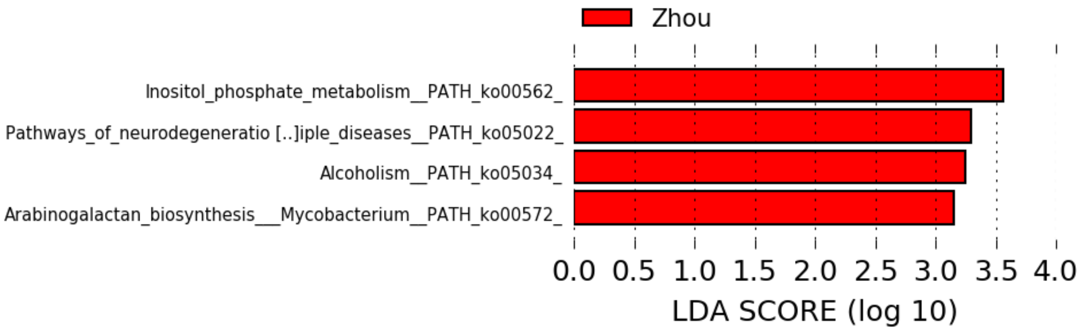

Figure S20. Functional LEfSe (case v. case), Studies A-C

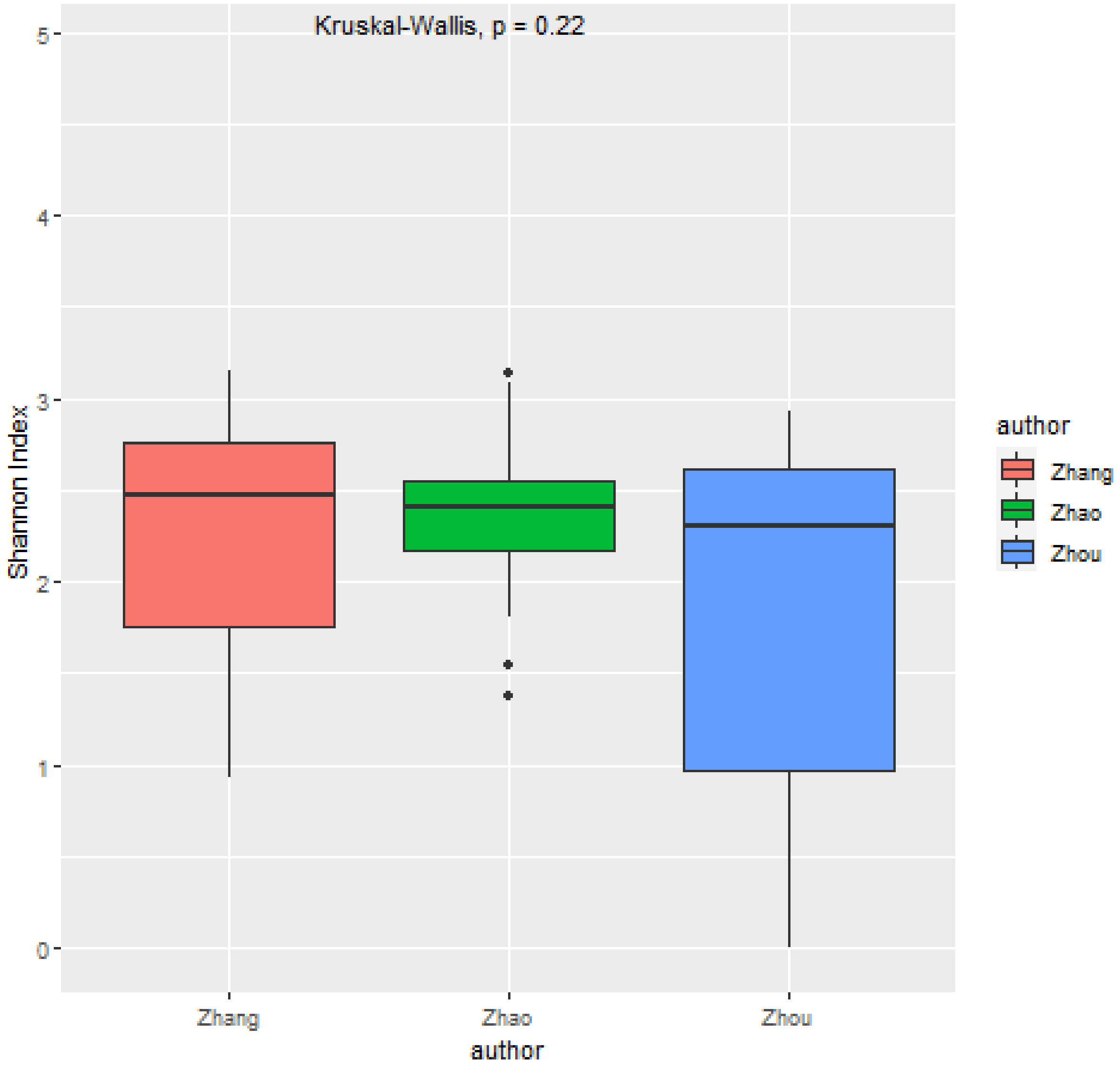

Figure S21. Shannon index comparison (control v. control) - Studies A-C

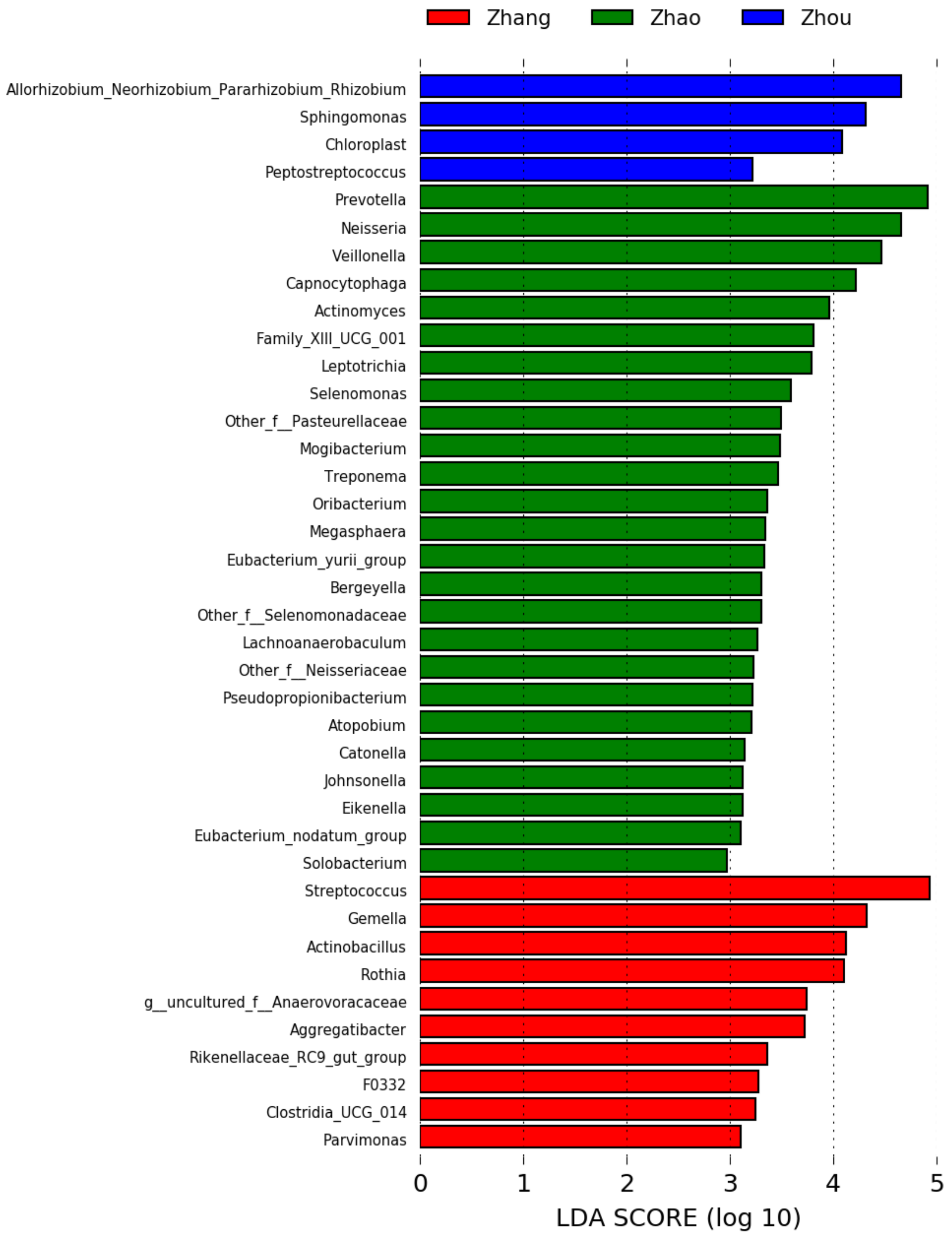

Figure S22. Taxonomic LEfSe (control v. control) - Studies A-C

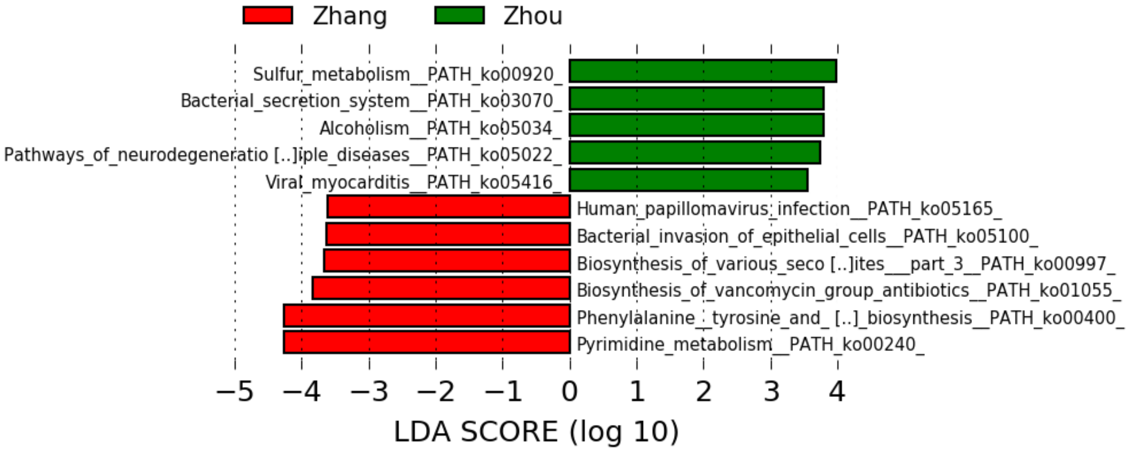

Figure S23. Functional LEfSe (control v. control) - Studies A-C
